# Fibrinogen αC‐regions are not directly involved in fibrin polymerization as evidenced by a “Double‐Detroit” recombinant fibrinogen mutant and knobs‐mimic peptides

**DOI:** 10.1111/jth.14725

**Published:** 2020-01-29

**Authors:** Cédric Duval, Aldo Profumo, Anna Aprile, Annalisa Salis, Enrico Millo, Gianluca Damonte, Julia S. Gauer, Robert A. S. Ariëns, Mattia Rocco

**Affiliations:** ^1^ Leeds Thrombosis Collective Discovery and Translational Science Department Leeds Institute of Cardiovascular and Metabolic Medicine University of Leeds Leeds UK; ^2^ Biopolimeri e Proteomica IRCCS Ospedale Policlinico San Martino Genova Italy; ^3^ Department of Experimental Medicine Center of Excellence for Biomedical Research (CEBR) University of Genova Genova Italy

**Keywords:** fibrin, fibrinogen, mutation, polymerization, thrombin

## Abstract

**Background:**

Fibrin polymerization, following fibrinopeptides A and B (FpA, FpB) cleavage, relies on newly exposed α‐ and β‐chains N‐termini (GPR, GHR; *A‐*, *B*‐knobs, respectively) engaging preexistent *a* and *b* pockets in other fibrin(ogen) molecules' γ‐ and (B)β‐chains C‐terminal regions. A role for mostly disordered (A)α‐chains C‐terminal regions “bridging” between fibrin molecules/fibrils has been proposed.

**Objectives:**

Fibrinogen Detroit is a clinically observed mutation (AαR19 → S) with nonengaging GPS *A*‐knobs. By analogy, a similar Bβ‐chain mutation, BβR17 → S, should produce nonengaging GHS *B*‐knobs. A homozygous “Double‐Detroit” mutant (AαR19 → S, BβR17 → S; DD‐FG) was developed: with *A*‐*a* and *B*‐*b* engagements endogenously blocked, other interactions would become apparent.

**Methods:**

DD‐FG, wild‐type recombinant (WT‐FG), and human plasma (hp‐FG) fibrinogen self‐association was studied by turbidimetry coupled with fibrinopeptides release high‐performance liquid chromatography (HPLC)/mass spectrometry analyses, and by light‐scattering following size‐exclusion chromatography (SE‐HPLC).

**Results:**

In contrast to WT‐FG and hp‐FG, DD‐FG produced no turbidity increase, irrespective of thrombin concentration. The SE‐HPLC profile of concentrated DD‐FG was unaffected by thrombin treatment, and light‐scattering, at lower concentration, showed no intensity and hydrodynamic radius changes. Compared with hp‐FG, both WT‐FG and DD‐FG showed no FpA cleavage difference, while ~50% FpB was not recovered. Correspondingly, SDS‐PAGE/Western‐blots revealed partial Bβ‐chain N‐terminal and Aα‐chain C‐terminal degradation. Nevertheless, ~70% DD‐FG molecules bearing (A)αC‐regions potentially able to associate were available. Higher‐concentration, nearly intact hp‐FG with 500‐fold molar excess GPRP‐NH_2_/GHRP‐NH_2_ knobs‐mimics experiments confirmed these no‐association findings.

**Conclusions:**

(A)αC‐regions interactions appear too weak to assist native fibrin polymerization, at least without knobs engagement. Their role in all stages should be carefully reconsidered.

**Essentials**

- αC‐regions are thought to actively complement knob‐hole interactions during fibrin assembly.
- Defective knobs in a recombinant “Double Detroit” mutant (DD‐FG) should impede fibril formation.
- Thrombin‐treated DD‐FG alone or fibrinogen with knobs‐mimics showed a total lack of associations.
- A more passive role of the α‐chains C‐terminal regions in fibrin assembly is proposed.

## 1 INTRODUCTION

Fibrinogen is a central player in blood coagulation, with important roles in pathological situations such as thrombosis,^1^, ^2^ atherosclerosis,^3^, ^4^ and cancer metastasis.^5^ It is a high‐molecular‐weight (~340 000 Da), elongated (~45 nm) glycoprotein circulating as an inactive precursor in the blood at ~3 to 5 mg/mL.^6^ Fibrinogen is composed of two pairs of three polypeptide chains, Aα, Bβ, and γ (Aα_2_Bβ_2_γ_2_; in human form 610, 461, and 411 amino acids, respectively^7^). All chains' N‐terminal ends are bundled together by S‐S bridges in a central “E‐region,” from which two triple coiled‐coil connectors depart in opposite directions, each held in register by two disulfide rings between the Aα‐, Bβ‐, and γ‐chains.^8^ At the end of the connectors, the Bβ‐ and γ‐chain C‐terminal parts form two outer D‐regions, within which each chain folds independently.^9^ Instead, the >400 C‐terminal residues of the Aα chains first reverse direction forming a fourth strand up to about halfway on the coiled‐coils connectors,^10^ and then protrude as mainly disordered appendages (“AαC‐regions”),^11^ within which a small partially ordered subdomain (Aα425‐503 in the human sequence) has been identified.^12^, ^13^, ^14^

Thrombin converts fibrinogen into a reactive species by cleaving two pairs of short peptides, called fibrinopeptides A and B (FpA and FpB, 16 and 14 residues, respectively), from the N‐termini of the Aα and Bβ chains in the central E‐region, generating the α_2_β_2_γ_2_ fibrin monomer.^15^, ^16^, ^17^ The resulting N‐termini in the α‐ and β‐chains, with initial sequence GPR and GHR, are called the *A* and *B* “knobs,” respectively.^18^ They engage very tightly, mainly by electrostatic interactions, into pre‐existing and readily available *a* and *b* “holes” in the D‐region's C‐terminal parts of the γ‐ and (B)β‐chains, respectively, in other fibrin(ogen) molecules.^18^ Rapid polymerization ensues, first forming elongated (proto)fibrils,^19^, ^20^ which by subsequent branching and lateral aggregation give rise to a three‐dimensional network, the clot scaffold that stabilizes the initial platelet plug during blood coagulation (see^7^, ^17^). FpA release is the key initial event, with *A*‐a interactions governing (proto)fibril formation in a final half‐staggered, double‐stranded arrangement.^20^, ^21^ FpB is released by thrombin later in the process, and the *B*‐*b* engagement enhances the lateral thickening of the fibers.^22^, ^23^ There is also evidence of promiscuity between the *A* and *B* knobs toward the *a* and *b* holes, probably derived from the common evolutionary origin of the fibrinogen chains.^24^

Several important aspects of fibrin polymerization have been elucidated over the years, but some key questions still remain. In particular, it has been proposed that the (A) αC‐regions interact with each other, and with the central E‐region in the fibrinogen molecule, and that they are released following fibrinopeptide cleavage, more likely after FpB removal.^11^, ^23^ The released αC‐regions have been postulated to assist fiber assembly by intermolecular binding between parallel protofibrils.^25^, ^26^ However, proving this αC‐regions release mechanism at the level of individual fibrin molecules is difficult, as they rapidly polymerize, and only large amounts of knobs‐mimic peptides inhibitors such as GPRP‐NH_2_ and GHRP‐NH_2_ (at ≥500‐fold molar ratio) can block this process.^27^ Because the *B*‐*b* engagement induces changes in the relative orientation of the β‐ and γ‐chains C‐terminal subdomains,^28^ binding of knobs‐mimics can have difficult to evaluate consequences at a structural level. However, they could still be employed to reveal other potential interactions between fibrin(ogen) molecules.

Among the many clinically observed fibrinogen mutations affecting fibrin formation (http://site.geht.org/base-fibrinogene 29), fibrinogen Detroit (AαR19 → S)^30^ is of particular interest. In this mutant, FpA can be cleaved by thrombin, but the resulting mutated *A*‐knob, GPS, is unable to bind either the *a* or *b* holes, leading to severely impaired fibrin formation, only partially rescued by the GHR normal *B*‐knobs binding to their cognate *b* holes.^31^ On this basis, we hypothesized that a similar mutation in the *B*‐knob, BβR17 → S, would stop it binding to either holes *b* or *a*. A mutant carrying both AαR19 → S and BβR17 → S substitutions should therefore reveal any other potential interaction between fibrin monomers following cleavage of both fibrinopeptides.

Here we report the development of this recombinant human fibrinogen mutant, that we have termed Double‐Detroit fibrinogen (DD‐FG), and its characterization before and after thrombin treatment. As it unfortunately sometimes happens with recombinant fibrinogen production in mammalian cells, we have encountered degradation issues with the DD‐FG mutant and wild‐type fibrinogen (WT‐FG), despite the addition of protease inhibitors during the purification procedures. This resulted in cleavage of portions of the AαC‐regions and of the first ~50N‐terminal amino acids of the Bβ‐chain, in a manner reminiscent of the formation of the so‐called fragment X by plasmin action.^32^ Nevertheless, the amount of intact or just slightly degraded species was sufficient to allow clear‐cut results to be obtained. Namely, we found that, despite thrombin cleavage of the fibrinopeptides, DD‐fibrin monomers showed no signs of polymerization whatsoever, neither by turbidity analysis, nor by time‐resolved static and dynamic light scattering. Similar results were obtained with a fibrinogen fraction with mostly intact AαC‐regions and in the presence of a large excess of both GPRP‐NH_2_ and GHRP‐NH_2_. Overall, these data failed to reveal any contribution of the αC‐regions, while confirming the fundamental role of knob‐hole interactions in powering fibrin polymerization. DD‐FG will also provide an essential new tool for the study of the molecular properties of fibrin monomers after their generation from fibrinogen by thrombin, without the interference of polymerization or the formation of a clot.

## 2 MATERIALS AND METHODS

### 2.1 DD‐FG and WT‐FG expression, purification, and quality control

Recombinant human AαR19S/BβR17S fibrinogen (DD‐FG) and WT‐FG were prepared as previously described.^33^ Detailed protocols, including for the enzyme‐linked immunosorbent assay tests, can be found in the Appendix S1. Final concentrations were determined spectrophotometrically at *λ* = 280 nm (ε^280^ = 1.51 mL mg^−1^ cm^−1^)^34^ and the purity of each recombinant fibrinogen batch was assessed by SDS‐PAGE under reducing conditions using 10% polyacrylamide (PAA) gels.^35^ Further characterization was conducted by Western‐blot analysis after SDS‐PAGE, using the mouse monoclonal antibody Y18 specific for the N‐terminal end of the Aα‐chains,^36^ and a rabbit polyclonal antibody against the C‐terminal 250‐491 region of the Bβ‐chains (Ab137830, Abcam, Prodotti Gianni). Color was developed with horseradish peroxidase‐conjugated goat anti‐mouse IgM (A‐8766, Sigma‐Aldrich) and anti‐rabbit IgG (7074S, Cell Signaling Technology, EuroClone) secondary antibodies, respectively, and 4‐chloro‐1‐naphthol (Sigma‐Aldrich) as a substrate. Dual color, Precision Plus recombinant molecular weight standards (161‐0374, Bio‐Rad) were used as markers. Quantification of the relative amounts of the Aα‐ and Bβ‐chains in the Western blots was done essentially as previously reported^35^ (for details, see the Appendix S1).

### 2.2 Turbidity coupled to fibrinopeptides release assays

These experiments were conceived to simultaneously monitor turbidity in a spectrophotometer and fibrinopeptide release by HPLC on the same sample, the latter at long time‐points. In addition to WT‐FG and DD‐FG, fibrinogen purified from human plasma (hp‐FG; type FIB3, Enzyme Research Laboratories) was used. An hp‐FG fraction with mostly intact AαC‐regions (HMW‐FG) was prepared as previously described,^35^, ^37^, ^38^ and used to perform experiments in the absence/presence of knobs‐mimic peptides GPRP‐NH_2_ (H‐1998, Bachem) and GHRP‐NH_2_ (synthesized in‐house, see Appendix S1). All experiments were performed in TBS buffer [tris(hydroxymethyl)aminomethane 50 mmol/L, NaCl 100 mmol/L, aprotinin 1 KIU/L, pH 7.4]. Human α‐thrombin was from Enzyme Research Laboratories (3081 NIHu/mg). One vial containing nominal 1000 NIHu was reconstituted with 1 mL of MilliQ water, and vials containing 20‐μL aliquots were quick‐frozen in liquid N_2_ and stored at −80°C. Each time an aliquot was used, its activity was carefully determined as detailed in the Appendix S1. A DU‐640 spectrophotometer with a thermostatted 6‐position sample changer (Beckman Coulter) and a thermostatting block (Thermomixer Comfort; Eppendorf) were used for the turbidity experiments and for the parallel sample incubation, followed by reverse‐phase HPLC (RP‐HPLC) and mass spectroscopy (MS) analyses. Quantitative analyses were performed, determining the areas for each fibrinopeptide peak using a skewed Gaussian function (EMG + GMG). Samples were checked by SDS‐PAGE before and after these turbidity experiments. Detailed protocols are provided in the Appendix S1.

### 2.3 Size‐exclusion chromatography

Size‐exclusion chromatography (SE‐HPLC) was performed both analytically, to check for the presence of high‐ and low‐molecular weight components in the fibrinogen preparations, and in a semipreparative way to isolate sufficient monomeric fractions for the static and dynamic light scattering experiments (see Appendix S1).

### 2.4 Static and dynamic light scattering

A 1999 static/dynamic light scattering (SLS/DLS) instrument (Protein Solutions DynaPro model 99E; Rheometric Scientific), equipped with a square 3‐mm path length, 40‐μL minimum volume quartz cuvette, was used. The DynaPro has a 50‐mW, *λ* = 824.8 nm solid‐state diode laser, and LS collection at a single 90° scattering angle is done in photon‐counting mode. Basic LS theory, data analysis, and a detailed protocol for all steps used in the preparation of working solutions are reported in the Appendix S1.

## 3 RESULTS

### 3.1 Characterization of recombinant and plasma‐derived fibrinogens

Reduced samples of the proteins were run on SDS‐PAGE, and were found to be consistent with apparently pure preparations, with bands corresponding to the standard Aα, Bβ, and γA chains (Figure S1A). However, a more detailed Western blot analysis using a monoclonal antibody specific for the N‐terminal end of the Aα‐chain revealed that in both WT‐ and DD‐FG, up to ~70% of the Aα chains presented varying levels of degradation in the AαC region (Figure S2 and Table S2). Furthermore, from the analyses of a Western‐blot stained with a polyclonal antibody against the C‐terminal region of the Bβ‐chain, two groups of bands could be discerned (Figure S3 and Table S3). The constituents of the first group had approximate molecular weights close to that of intact Bβ chains with up to two sialic acids in its single carbohydrate chain (theoretical 53 900‐54 450), whereas the second group could result in the hp‐FG sample by the loss of the N‐terminal 1‐42 residues (mol. wt. ~4600), a classic plasmin‐degradation event.^39^ The corresponding DD‐FG and WT‐FG bands in this second group appeared to have slightly higher molecular weights (Table S3), suggesting that a different process might have generated a similar N‐terminal degradation of the Bβ chains. In these particular batches analyzed, about 25% (DD‐FG) and 30% (WT‐FG) of the Bβ chains appeared missing the N‐terminal residues (Table S3), which include the FpB and the *B*‐knob residues. Given the size shift, it is unlikely that the proteolysis took place at the C‐terminal end. Attempts to prevent this degradation with additional protease inhibitors have so far been unsuccessful, suggesting that a different cell line or expression system might be necessary in the long run to obtain more pristine products. However, the main self‐interaction domain within the (A)αC‐regions has been identified within residues Aα425‐503.^14^ Because the Aα1‐503 stretch has a predicted molecular weight of 54 589, this value was used as a cutoff to conservatively calculate from the SDS‐PAGE/Western blot data (Table S2) the percentage of molecules bearing AαC regions potentially able to interact. About 70% of DD‐FG molecules (and ~50% of both hp‐FG and WT‐FG) were found to contain the AαC self‐interaction region, allowing us to perform meaningful polymerization studies. Indeed, in initial assays at both low and high thrombin concentrations, WT‐FG behaved as a typical fibrinogen sample, whereas DD‐FG did not show any increase in turbidity (Figure S1B). In addition, preliminary fibrinopeptide release experiments indicated cleavage of both FpA and FpB from the recombinant FGs (data not shown).

### 3.2 Turbidity coupled to Fps release studies

**Figure 1:**
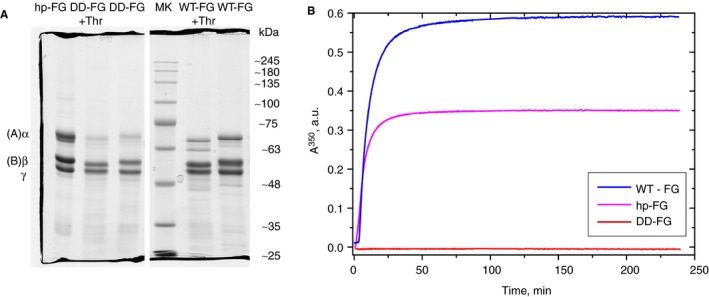
Polymerization kinetics of fibrinogen preparations. (A) SDS‐PAGE on a 10% PAA gel under reducing conditions (two portions of the same gel) of control samples (hp‐FG, DD‐FG, and WT‐FG), samples from the turbidity experiment after thrombin (Thr) addition (DD‐FG + Thr and WT‐FG + Thr), and a molecular weight marker (MK). The normal FG chains positions are indicated on the left side; the standards molecular weights listed on the right side are only indicative. (B) turbidity time course of WT‐FG (blue trace), hp‐FG (magenta trace), and DD‐FG (red trace), all at 0.3 mg/mL, after activation with thrombin at 0.08 NHIu/mL (0.28 NIHu/mg FG)

After prolonged treatment with thrombin (see below), both WT‐FG and DD‐FG showed a complete, small but noticeable reduction in the sizes of both the Aα‐ and Bβ‐chains, attributable to normal cleavage of FpA and FpB (Figure 1A). Although both hp‐FG and WT‐FG displayed a typical turbidity profile,^40^ no change whatsoever was observed in absorbency for DD‐FG (Figure 1B). The differences in the profiles and final turbidity levels for the hp‐FG and WT‐FG likely derived from the relative purity of the two samples leading to differences in clot structure. For instance, whereas recombinant WT‐FG is almost devoid of aggregates, these are usually present in hp‐FG, and reduce final fiber thickness.^41^, ^42^ In Figure S4, turbidity profiles obtained at the same final fibrinogen concentration and thrombin activity are shown for the same hp‐FG sample before and after SE‐HPLC treatment (see the following section). For the turbidity experiments reported in Figure 1B, aggregates were not removed from our hp‐FG samples.

**Figure 2:**
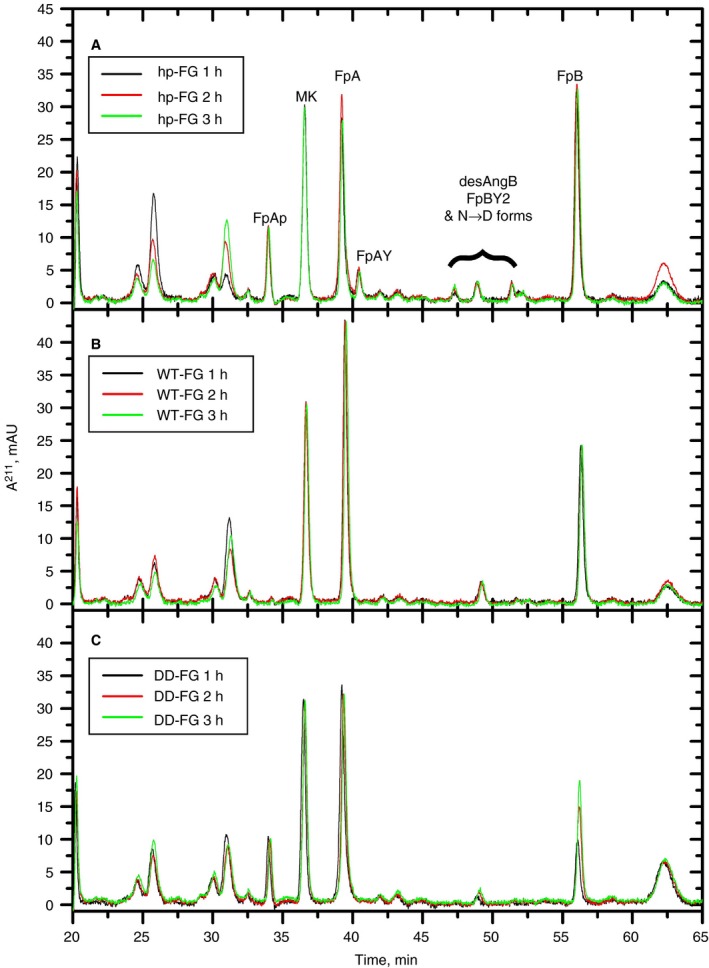
RP‐HPLC fibrinopeptides release analyses following turbidity experiments with various fibrinogen preparations. hp‐FG (A), WT‐FG (B), and DD‐FG (C), all at 0.3 mg/mL, after thrombin activation (0.28 NIHu/mg FG) at 25°C for 1 hour (black traces), 2 hours (red traces), and 3 hours (green traces). All data shown here are blank‐subtracted. Injection volumes were all 20 μL, except 15 μL for the DD‐FG 1‐hour sample, whose A^211^ values were therefore rescaled by a 4/3 factor. The fibrinopeptides are identified in (**A**) (FpAP, phosphorylated FpA; MK, marker peptide; FpAY, FpA lacking the N‐terminal A residue; desArgB, FpB lacking the C‐terminal R residue; FpBY2, FpB lacking the N‐terminal pyro‐N and G residues; N → D, deamidated forms of all FpBs; see^55^)

The release of fibrinopeptides was analyzed by RP‐HPLC in parallel to the turbidity experiments (Figure 2). For all datasets, complete superimposition of a marker (MK) peak at the three incubation times was observed (Figure 2A‐C), suggesting that no material was differentially lost in the boiling/filtration/injection steps. All non‐labeled peaks present were found not to be derived from known or new fibrinopeptide species by MS‐MS analyses (data not shown).

For hp‐FG, (Figure 2A) there was no difference in the heights/shapes of all peaks as a function of the incubation time with thrombin, indicating that after 1 hour the fibrinopeptide release had already reached plateau.

For WT‐FG (Figure 2B) the FpAP and FpAY peaks were practically absent, as previously noted with recombinant fibrinogens expressed in CHO cells.^43^ As a result, the FpA peak was higher than for hp‐FG. Only desArgB appeared to be present among the FpB variants, but the FpB peak was noticeably lower than its counterpart from hp‐FG. Similar to hp‐FG, the release of all fibrinopeptides for WT‐FG appeared complete after 1‐hour incubation with thrombin.

The DD‐FG fibrinopeptides analyses showed some differences (Figure 2C). FpAP appeared to be present, confirmed by MS‐MS analyses (data not shown), and FpB was apparently released more slowly, with the 2‐hour thrombin incubation peak still being lower than that observed after 3 hours.

Approximate absolute amounts of fibrinopeptides were then determined for the 3 hours timepoints, using calculated ε^211^ molar extinction coefficients (see Appendix S1), as shown in Table 1. Given the low variability of the MK areas, within ±2% of their weighted mean (11.358 ± 0.012 mAU), data normalization was unnecessary. The amounts determined for each fibrinopeptide species were then grouped into total FpA and FpB, allowing to calculate their ratio. The recovery of total FpA, based on the theoretical injected amounts of 36 pmol, was ~90% for all three fibrinogens used. Considering the uncertainties in the calculated ε^211^ values, this result nicely supports complete cleavage of FpA from all our samples.

**Table 1:** Areas and derived amounts (pmol) from the A^211^ of released fibrinopeptides individual peaks, with the totals for FpA and FpB species and their ratio

Peak	Sample	hp‐FG		WT‐FG		DD‐FG	
	Calc. ε^211^ (M^−1^ cm^−1^)	Area (mAU × min)	Amount (pmol)	Area (mAU × min)	Amount (pmol)	Area (mAU × min)	Amount (pmol)
MK (% from wm)	ND	11.17 ± 0.05 (−1.7 ± 0.4)	ND	11.53 ± 0.02 (+1.5 ± 0.2)	ND	11.30 ± 0.02 (−0.5 ± 0.2)	ND
FpA	24 263	10.29 ± 0.02	21.20 ± 0.04	15.73 ± 0.02	32.42 ± 0.04	12.85 ± 0.02	26.49 ± 0.04
FpAP	24 263	3.48 ± 0.02	7.17 ± 0.03	nd	nd	2.83 ± 0.02	5.84 ± 0.03
FpAY	22 957	1.78 ± 0.02	3.88 ± 0.04	nd	nd	nd	nd
FpB	29 317	13.12 ± 0.02	22.38 ± 0.03	9.90 ± 0.02	16.88 ± 0.04	7.31 ± 0.03	12.47 ± 0.04
desArgB	27 135	1.36 ± 0.02	2.51 ± 0.03	1.63 ± 0.02	3.00 ± 0.04	0.84 ± 0.02	1.55 ± 0.03
FpB var1	29 317	0.96 ± 0.02	1.64 ± 0.03	nd	nd	nd	nd
FpB var2	29 317	0.88 ± 0.25	1.51 ± 0.42	nd	nd	nd	nd
FpB var3	29 317	0.94 ± 0.25	1.60 ± 0.42	nd	nd	nd	nd
Total FpA	na	ND	32.25 ± 0.06	ND	32.42 ± 0.04	ND	32.32 ± 0.05
Total FpB	na	ND	29.63 ± 0.60	ND	19.88 ± 0.06	ND	14.02 ± 0.05
FpA/FpB	na	ND	1.09 ± 0.02	ND	1.63 ± 0.01	ND	2.31 ± 0.01

Injected samples were 20 μL each of hp‐FG, WT‐FG, and DD‐FG at 0.3 mg/mL after 3 h thrombin incubation at 0.28 NIHu/mg FG in TBS‐PEG followed by 1 min boiling and filtration.

Abbreviations: na, not applicable; nd, not detected; ND, not done; wm, weighted mean.

For hp‐FG, the FpA/FpB ratio was close to 1 (Table 1), as expected. However, for both WT‐FG and DD‐FG, the FpA/FpB ratio was higher (~1.6 and ~2.3, respectively; Table 1), indicating that about one‐third and one‐half of FpB, respectively, were not recovered. By careful MS‐MS analysis of all peaks present in our chromatograms, no alternative FpB form that could account for the missing amounts was found. Moreover, by SDS‐PAGE analyses (Figure 1A) all FpB is removed from the Bβ‐chains, excluding the presence of noncleavable FpB in the recombinants. The Western blot analyses could provide only a partial explanation. As shown in Table S3, ~31% and ~26% of the intact Bβ‐chains were found missing in these WT‐FG and DD‐FG preparations, respectively. For WT‐FG, this was reasonably close to the missing FpB amount in the HPLC analyses, whereas for DD‐FG another ~30% was unaccounted for. Unfortunately, the amount of DD‐FG sample taken from this experiment was insufficient for Western blot following the regular SDS‐PAGE analysis, and this particular material‐ and time‐consuming experiment was not repeated. It is conceivable, however, given the variability observed in the degradation of DD‐FG batches, that this batch had an even higher amount of N‐terminally cleaved Bβ‐chains. Although this issue is being further investigated, the combined results still support the notion that both Fps were cleaved from DD‐FG, and that the absence of clot formation was due to the defective, non‐binding *A* and *B* knobs.

Experiments with GPRP‐NH_2_ and GHRP‐NH_2_ knobs mimics were also conducted using hp‐FG. As shown in Figure S5, a 600 × molar excess of GPRP‐NH_2_ alone (magenta trace) greatly delayed but not completely abolished lateral aggregation, likely because of a “rescue” effect by the *B*:*b* engagement. When GHRP‐NH_2_ was also added in a 290 × molar excess (Figure S5, blue trace) no lateral aggregation took place. It was found, however, that while an excess of GPRP‐NH_2_ had no influence on FpA cleavage by thrombin, GHRP‐NH_2_ did delay FpB cleavage (data not shown). This effect could be overcome by a 10‐fold increase in thrombin concentration (data not shown).

### 3.3 SE‐HPLC and SLS/DLS studies

**Figure 3:**
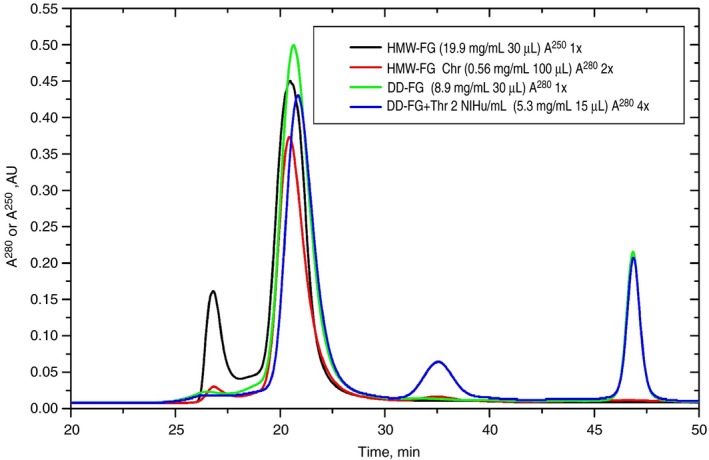
SE‐HPLC profiles of fibrinogen preparations. Concentrated HMW‐FG (black; injected 30 μL at 19.9 mg/mL), HMW‐FG peak fraction used for SLS/DLS (red; injected 100 μL at 0.56 mg/mL), concentrated DD‐FG (green; injected 30 μL at 8.9 mg/mL), and concentrated DD‐FG after incubation for 2 h with thrombin at 2 NIHu/mL (blue; injected 15 μL at 5.3 mg/mL). The absorbance was monitored at *λ* = 250 nm for the black trace, and at *λ* = 280 nm for all other traces. To facilitate comparison, the A^280^ values of the red and blue traces were blank‐subtracted, multiplied respectively 2 and 4 times, followed by blank readdition

Because turbidity mostly detects the lateral aggregation of fibrin fibers, the possibility that protofibrils and/or small oligomers could still form after thrombin activation of DD‐FG or of HMW‐FG in the presence of knobs‐mimics was investigated by SLS/DLS. To perform meaningful SLS/DLS measurements, aggregates and/or degradation products were removed by semi‐preparative SE‐HPLC. HMW‐FG, containing mostly intact Bβ chains and relatively intact Aα chains (~90% with Aα1‐503, see Figure S6) was first used, manually collecting the peak fraction starting and ending at approximately half of the peak intensity (~1 mL, 0.56 mg/mL). The HMW‐FG SE‐HPLC traces before and after chromatography (Figure 3; black and red traces, respectively) showed a strong reduction of the aggregates peak, as well as the absence of degradation products, with the main peak eluting at practically the same position (30.48 vs 30.43 minutes). More concentrated HMW‐FG and hp‐FG fractions were subsequently purified, both yielding ~0.7 mL at 0.9‐1.3 mg/mL (data not shown).

SE‐HPLC was then applied to DD‐FG, after concentration to ~9 mg/mL, yielding ~0.65 mL at 0.3 mg/mL. Its profile (Figure 3; green trace) showed that although aggregates were practically absent, a late‐eluting relatively intense peak was present. Because all subsequent experiments were performed on the main peak fraction, containing pure DD‐FG as ascertained by SDS‐PAGE, its nature was not further investigated. A small displacement of the top of the peak, 30.63 minutes, was observed compared to the HMW‐FG sample, possibly reflecting the loss of portions of the AαC regions as indicated by SDS‐PAGE/Western blot analyses (Table S2). Then, as a test of the lack of formation of oligomers even at high concentration, 10 μL of a 5 NIHu/mL thrombin solution in TBS were added to 15 μL of concentrated DD‐FG, bringing it to 5.3 mg/mL (thrombin final nominal activity 2 NIHu/mL, 0.37 NIHu/mg DD‐FG). After incubation for 2 hours at 25°C, 15 μL were then injected, without spin‐filtering, in the SE‐HPLC system. The resulting profile (Figure 3; blue trace) confirmed that no polymers were detectable by this method. The main peak eluted slightly later (30.86 minutes) than the untreated material. The late‐eluting peak was still present, as well as a new, unidentified one eluting in between, most likely coming from material present in the thrombin solution.

**Figure 4:**
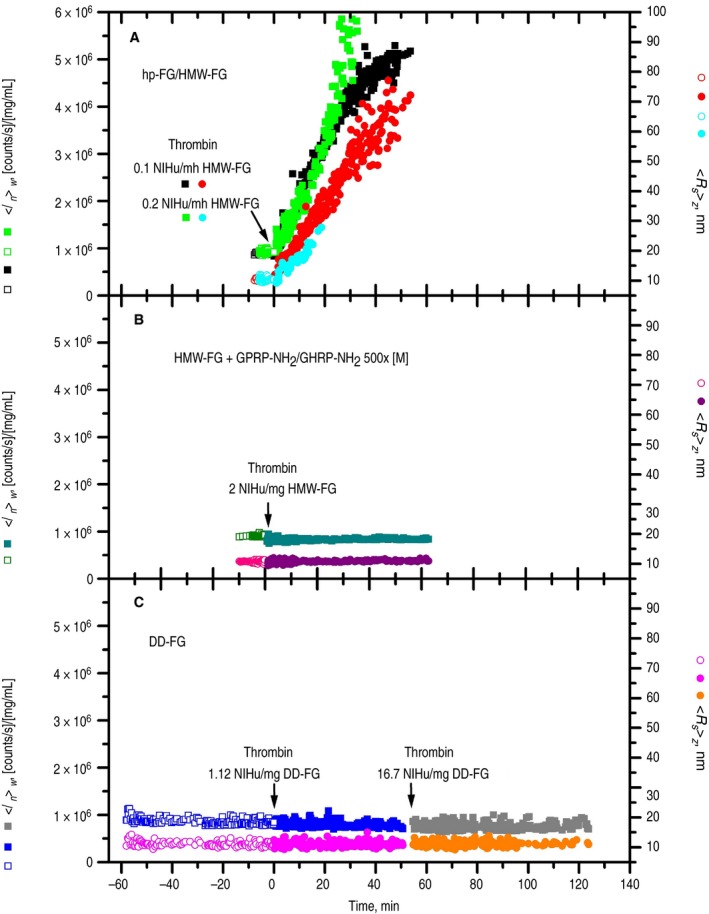
SLS/DLS experiments with fibrinogen preparations. (A) HMW‐FG and hp‐FG; (B) HMW‐FG with GPRP‐NH_2_/GHRP‐NH_2_ each 500× molar excess; (C) DD‐FG. The left *y*‐axes report the weight‐average intensities normalized by the sample concentration <*I_n_*>*_w_* ([counts/s]/[mg/mL]; squares), whereas the right *y*‐axes report the *z*‐average Stokes' radius <*R_s_*>*_z_* (nm; circles). Open symbols (negative time points), samples before thrombin addition; filled symbols, samples after thrombin addition (indicated by the labeled arrows). Data were mostly collected at 1‐second acquisition times (1 every 15‐20 points are shown for clarity), with 10‐ to 20‐second acquisition times also used in nonevolving regions; heavy “spikes” were manually removed. (A) 50 μL HMW‐FG at 0.517 mg/mL (black and red symbols) and hp‐FG at 0.22 mg/mL (green and cyan symbols) before thrombin addition (empty symbols), followed by HMW‐FG at 0.431 mg/mL after addition of 10 μL 0.23 NIHu/mL thrombin (final 0.1 NIHu/mg FG; filled black and red symbols) and by hp‐FG at 0.16 mg/mL after addition of 20 μL 0.11 NIHu/mL thrombin (final 0.2 NIHu/mg FG; filled green and cyan symbols). (B) 50 μL HMW‐FG at 0.83 mg/mL with GPRP‐NH_2_ and GHRP‐NH_2_ both 1.92 mmol/L (open dark green and pink symbols), followed by addition of 2 μL of 40 NIHu/mL thrombin (final HMW‐FG 0.8 mg/mL, thrombin 2 NIHu/mg FG; filled dark cyan and purple symbols). (C) 50 μL DD‐FG at 0.25 mg/mL before thrombin addition (open blue and magenta symbols) monitored for 1 h; at 0.18 mg/mL after addition of 20 μL 0.70 NIHu/mL thrombin (final 1.12 NIHu/mg FG, filled blue and magenta symbols), monitored for 50 minutes; and at nominal 0.167 mg/mL after addition of 5 μL 41.7 NIHu/mL thrombin (final 17.8 NIHu/mg FG, filled gray and orange symbols), monitored for 70 minutes

SLS/DLS studies were then performed on 50 μL each of the SE‐HPLC purified samples, devoid of any pre‐existing aggregates, before and after thrombin treatment. In Figure 4, the left *y*‐axis reports the SLS weight‐average intensity normalized by the sample concentration <*I_n_*>*_w_* ([counts/s]/[mg/mL]), whereas the right *y*‐axis reports the DLS‐derived *z*‐average Stokes' radius <*R_s_*>*_z_* (nm). The HMW‐FG samples, given their integrity, were first used to check the procedures and the quality of the measured molecular parameters. As shown in Figure 4A (black squares and red circles), data on untreated HMW‐FG at 0.517 mg/mL were first recorded (“negative” time points), followed by activation with 10 μL of a 0.23 NIHu/mL thrombin solution (thrombin final concentration 0.04 NIHu/mL, 0.1 NIHu/mg FG). Reasonably constant data were obtained during the ~10 minutes before thrombin addition (empty symbols), followed by a rapid increase thereafter (filled symbols), indicating polymerization. HMW‐FG at a higher concentration was also studied (0.91 mg/mL; data not shown), and the <*R_s_*>*_z_* results of several individual 20 seconds acquisitions were then averaged, reported in Table 2 as [<*R_s_*>*_z_*]*_wa_*. Table 2 also contains the *w*‐average intensities [<*I_bs_*>*_w_*]*_wa,n_*, (average blank‐subtracted before concentration normalization) together with the derived apparent molecular weights [<M*>*_w_*]*_wa_* and the [<*M* ^0^>*_w_*]*_wa_* values corrected for the known^43^ HMW‐FG concentration dependence (see Appendix S1). The HMW‐FG [<*R_s_*>*_z_*]*_wa_* is close to the accepted value for fibrinogen (10.4 nm, see^43^), demonstrating a practically monomeric sample prior to thrombin addition. This was confirmed by the derived [<*M* ^0^>*_w_*]*_wa_* in excellent agreement with that expected for HMW‐FG, ~333 000 g/mol.^43^ Both data confirmed optimal performance of the SLS/DLS set‐up.

**Table 2:** SOS‐weighted averages (*wa*) of the Stokes' radii [<*R_s_*>*_z_*]*_wa_*, of the blank‐subtracted and concentration normalized intensities [<*I_bs_*>*_w_*]*_wa,n_*, and of the derived apparent [<M*>*_w_*]*_wa_* and extrapolated to *c* = 0 [<*M* ^0^>*_w_*]*_wa_* molecular weights for HMW‐FG without and with peptide knobs‐mimics prior and after thrombin (Thr) addition, and for DD‐FG prior and after thrombin additions

	HMW‐FG	HMW‐FG + 500× [M] GPRP‐NH_2_/GHRP‐NH_2_	HMW‐FG + 500× [M] GPRP‐NH_2_/GHRP‐NH_2_ + Thr 2 NIHu/mg FG	DD‐FG	DD‐FG + Thr 1.12 NIHu/mg FG	DD‐FG + Thr 17.8 NIHu/mg FG
Acquisition time (s)	20	20	10	20	1	20
Data averaged	19	27	95	31	1610	23
*c* (mg/mL)	0.910	0.830	0.800	0.250	0.179	*0.167* a
[<*R_s_*>*_z_*]*_wa_* (nm)	10.81 ± 0.09^b^	10.79 ± 0.06	10.99 ± 0.25	11.27 ± 0.48^b^	11.42 ± 1.50	11.12 ± 0.45
[<*I_bs_*>*_w_*]*_wa,_*_n _[counts/s]/[mg/mL]	886 000 ± 14 000	873 000 ± 7000	759 000 ± 17 000	804 000 ± 33 000	757 000 ± 84 000	*617 000 ± 26 000* a
[<M*>*_w_*]*_wa_* g/mol	377 000 ± 6000	371 000 ± 3000	323 000 ± 7000	342 000 ± 14 000	322 000 ± 36 000	*262 000 ± 11 000* a
[<*M* ^0^>*_w_*]*_wa_* c g/mol	324 000 ± 5000	324 000 ± 2600	(323 000 ± 7000)	328 000 ± 13 000	(322 000 ± 36 000)	(*262 000 ± 11 000* a)

a Values in italics are likely affected by a relatively large uncertainty in the concentration value.

b The difference between these two values is statistically significant at the 99% confidence level (*P* < 0.001, one‐tailed Student *t *test).

c Values extrapolated to *c* = 0 using the second virial coefficient of HMW‐FG determined by Raynal et al.^43^ Values within round brackets were not extrapolated to *c* = 0 (see Appendix S1 for details).

An additional experiment was performed with the SE‐HPLC‐purified hp‐FG sample, after dilution to 0.22 mg/mL, similar to that of DD‐FG (see below ). As shown in Figure 4A (green squares and cyan circles), data practically superimposable with those of HMW‐FG were obtained before thrombin addition (empty symbols). Values of [<*R_s_*>*_z_*]*_wa_* = 10.4 ± 0.1 nm and [<*M* ^0^>*_w_*]*_wa_* = 375 000 ± 3000 g/mol confirmed that our samples mainly contained monomeric material. The polymerization was then initiated with thrombin at a final 0.2 NIHu/mg FG (Figure 4A, filled symbols): <*I_n_*>*_w_* and <*R_s_*>*_z_* data very similar to those of HMW‐FG were obtained up to ~20 minutes, becoming very noisy afterwards. A parallel turbidity test on this hp‐FG sample showed that little absorbance changes happened until ~10 minutes, starting to increase afterwards (see Figure S7). This indicates that data on both the HMW‐FG and hp‐FG samples were collected mainly during the polymerization lag time, well before any fiber thickening took place.^44^ This is confirmed by the terminal <*I_n_*>*_w_* value of ~5.2 × 10^6^ [(counts/s)/(mg/mL)] for HMW‐FG (Figure 4A) corresponding to a <M*>*_w_* of ~2.2 × 10^6^ g/mol (eg, see Fig. 2 in^45^).

The effect of knobs‐mimics was then studied. To 90 μL of the SE‐HPLC‐treated HMW‐FG sample, 2.2 and 3.8 μL of concentrated GPRP‐NH_2_ and GHRP‐NH_2_ were respectively added before centrifiltration (final 500 × molar excess). SLS/DLS was performed on 50 μL before (Figure 4B, empty dark green and pink symbols; HMW‐FG 0.83 mg/mL) and after addition of 1.7 μL 1.6 NIHu/mL thrombin (Figure 4B, filled dark cyan and purple symbols; final HMW‐FG 0.8 mg/mL, thrombin 2 NIHu/mg FG). No changes were observed up to 1 hour after thrombin addition (Figure 4B). The complete removal of all fibrinopeptides was ascertained both by SDS‐PAGE/Western blots (Figure S6) and RP‐HPLC (Figure S8). Before activation, the [<*R_s_*>*_z_*]*_wa_* and [<*M* ^0^>*_w_*]*_wa_* were indistinguishable from those of HMW‐FG without knobs‐mimics, and only a very small increase of [<*R_s_*>*_z_*]*_wa_* resulted at the end of thrombin action (Table 2). Interestingly, the terminal [<M*>*_w_*]*_wa_* value without extrapolation to *c* = 0 was close to the initial [<*M* ^0^>*_w_*]*_wa_*, suggesting that removal of the fibrinopeptides practically abolished intermolecular unspecific interactions.

Experiments with the SE‐HPLC purified DD‐FG sample are reported in Figure 4C, where a 0.25 mg/mL solution was first monitored for ~1 hour (open blue and magenta symbols), and then thrombin was added (final 0.20 NIHu/mL, 1.120 NIHu/mg FG) with the resulting solution monitored for ~50 minutes (filled blue and magenta symbols). A higher thrombin concentration than those used for HMW‐FG and hp‐FG was used to avoid long incubation times. At the end of this period, an even more concentrated thrombin solution was added (final ~3 NIHu/mL, ~18 NIHu/mg FG), and the resulting solution was monitored for another ~70 minutes (filled gray and orange symbols). Figure 4C clearly shows that there was no significant change after thrombin treatment in either the normalized SLS intensities, nor in the DLS‐derived <*R_s_*>*_z_*. As for the molecular parameters, the data collected in Table 2 first established that the initial [<*M* ^0^>*_w_*]*_wa_* was nearly identical to that of HMW‐FG. There was, however, a small but significantly (*P* = .001) higher value, 11.27 ± 0.48 nm, of the initial DD‐FG [<*R_s_*>*_z_*]*_wa_* compared to HMW‐FG. This could be related to conformational differences between HMW‐FG and the DD‐FG sample, perhaps due to the missing (B)β‐chain N‐terminal residues and their interaction with the αC‐regions. Interestingly, this difference was practically maintained after each thrombin addition (Table 2). Again, the observed decrease of [<*I_bs_*>*_w_*]*_wa,n_* and its derived [<M*>*_w_*]*_wa_* confirms the abolition of unspecific intermolecular interactions after fibrinopeptide removal. The very low [<M*>*_w_*]*_wa_* value obtained after the second thrombin addition could instead result from an overestimation of final sample concentration. If anything, this decrease still reinforces the notion that no polymerization whatsoever took place in the DD‐FG solution following fibrinopeptides removal by the relatively high thrombin concentrations employed.

## 4 DISCUSSION

We have reported here the development of a novel recombinant fibrinogen, in which both knobs are mutated to prevent fibrin polymerization. The design of this recombinant fibrinogen was inspired by fibrinogen Detroit, which bears a mutation in knob *A* and shows reduced polymerization. By analogy, we generated a Double‐Detroit fibrinogen, with both the *A* and *B* knobs mutated. Although other naturally occurring mutations leading to unproductive *A* knobs have been reported (e.g., München, AαR19 → D,^46^ or Aarhus, AαR19 → G^47^), we considered the R → S substitution as the most likely to produce a similar effect on the *B* knob. We have shown that these *A* and *B* knob mutations when combined completely abolish protofibril formation, lateral aggregation, and the formation of a polymeric fibrin network. While these experiments were conducted at a relatively low DD‐FG concentration (~0.2‐0.3 mg/mL), the SE‐HPLC test we performed after thrombin treatment of a much more concentrated DD‐FG sample (~5 mg/mL, at the upper end of the physiological range^6^) still did not produce any evidence of polymer formation. At the very least, if putative complexes were dissociating by dilution during elution, a substantially altered peak shape should have been observed.

Concerning the observed Bβ‐chain N‐terminal degradation, more than ~50% DD‐FG molecules in our samples would have at least a *B*‐knob available to lead to polymer formation, which was undetectable by both the high‐sensitivity light scattering experiments and the overall turbidity measurements. In addition, the fact that all FG preparations showed similar Bβ‐chain degradation profiles allowed for their direct comparison. As for the AαC region degradation, ~70% of the DD‐FG molecules in our samples would bear the Aα425‐503 self‐interacting domain identified within the (A) αC regions.^14^ Importantly, the observed degradation patterns are also common in fibrinogen from human plasma.^48^

These experiments were complemented by nearly intact HMW‐FG at a substantially higher concentration treated with thrombin in the absence or presence of the peptide knobs‐mimics GPRP‐NH_2_ and GHRP‐NH_2_. Again, we failed to observe any evidence of complex formation both by turbidity and SLS/DLS experiments.

Interestingly, in both DD‐FG and HMW‐FG samples with knobs mimics, nonspecific intermolecular FG‐FG interactions seemed to disappear upon thrombin treatment. Recombinant α251‐FG, lacking the Aα252‐610 C‐terminal region, has a similar absence of non‐specific interactions even without fibrinopeptides removal.^43^ Because at pH 7.4 a reduction of net charge from −22 to −12 happens upon fibrinopeptides removal, and α251‐FG has a net charge of −18, perhaps a common charge‐related mechanism is responsible for this effect.

A considerable body of evidence exists regarding the proposed involvement of the AαC‐regions in “helping” fibrin assembly at certain stages (early reviews^49^, ^50^). First, electron microscopy studies suggested a “release” mechanism following fibrinopeptide cleavage based on differences in the (A)αC‐regions location between not‐cleaved and enzyme‐treated fibrinogen.^11^, ^25^ Additional studies showed concentration‐dependent aggregation of recombinant αC‐region fragments,^14^ and optical tweezers experiments demonstrated αC‐region interactions with the central E‐region and, more weakly, between them.^51^ More recently, AFM and AFM/turbidity studies further investigated the issue.^52^, ^53^ The consensus picture was that the αC‐regions following FpB cleavage extend further and help the lateral aggregation of fibrils by binding to each other. Furthermore, a very recent molecular dynamics simulation^54^ investigated the role of a particular residue, AαM476, located in the β‐hairpin present within the only (partially) structured domain so far identified in the αC‐region.

However, the most sensitive SLS/DLS experiments presented here did not show any changes in the aggregation status of DD‐FG samples following thrombin treatment. Based on the published *K_d_* for the recombinant human fibrinogen αC‐region (α392‐610), 12 μmol/L,^14^ we can calculate (see Appendix S1) that for HMW‐FG with knobs mimics and for DD‐FG, 13% and 3% dimers should have respectively formed following prolonged thrombin treatment. To ascertain what level of stable complexes our SLS/DLS experiments would have been able to reveal, we have performed calculations of the predicted weight‐average molecular weight <*M*>*_w_* as a function of the percentage of hypothetical αC‐mediated dimers. As shown in Tables S4 and S5, although we should have just barely detected 3% dimers in activated DD‐FG solutions (*P* = .03, 95% CI), we should have definitively seen the effect of 13% dimers (*P* < .00001, 95% CI) in HMW‐FG solutions with peptide knobs mimics. These calculations suggest that interactions between αC‐regions in native fibrin(ogen), or any other interactions between fibrin molecules, are too weak to be able to lead to any assembly following fibrinopeptide cleavage in the absence of knob‐hole engagement.

A possible explanation that will reconcile our findings with the existence of αC‐αC interactions, is to reverse the logic behind the currently accepted mechanistic view. That is, it could be conceivable that it is the lateral thickening of the fibrils that brings the αC‐regions in sufficiently close proximity to each other and allows their reciprocal binding. This will allow immediate reinforcement of the fibers in terms of mechanical strength and resistance to proteolysis, both of which are later further enhanced by factor XIIIa‐mediated crosslinking. As for what regulates the dramatic fiber thickening that follows the fibrin assembly lag phase, other mechanisms could be prevalent, from the change in the D‐regions/coiled‐coils relative orientation following *B*‐*b* engagement,^28^ to the collapse of hyperbranched fibrils,^43^ or their combination. Clearly, more work is necessary to better understand this mechanism.

In conclusion, overall, our data strongly support that formation of the fibrin clot is critically dependent only on the residues residing in the *A‐* and *B*‐knobs that are exposed after thrombin cleavage of fibrinogen. Although we cannot exclude that undetected issues could affect the recombinant fibrinogens behavior, it is the combined results obtained with DD‐FG, having similar degradation as “normal” plasma fibrinogen, and with HMW‐FG plus knobs‐mimics, with nearly intact Aα‐ and Bβ‐chains, that preclude a relevant role for other interactions in fibrin formation. Importantly, the DD‐FG described in this study provides a novel crucial tool compound with which, once degradation issues are resolved, we will be able to study monomeric fibrin structural and functional properties, such as the proposed αC‐regions release and other conformational changes following thrombin treatment, in the absence of polymer formation, and in the absence of peptide mimics to bind the polymerization pockets, which by themselves may affect the fibrin monomer conformation.

## CONFLICT OF INTERESTS

All authors declare no conflicts of interest.

## ADDENDUM

R.A.S. Ariëns and M. Rocco conceived the study, wrote the paper, and contributed equally; C. Duval prepared the mutant fibrinogen, performed experiments, and wrote the paper; J. Sandrin‐Gauer performed recombinant fibrinogen expression and purification; A. Profumo, A. Aprile, A. Salis, and M. Rocco performed experiments and analyzed data; E. Millo synthesized and analyzed peptides; G. Damonte provided equipment and assisted in data analysis.
