# Supplementary material for: Fibrinogen αC‐regions are not directly involved in fibrin polymerization as evidenced by a “Double‐Detroit” recombinant fibrinogen mutant and knobs‐mimic peptides
Source: J Thromb Haemost. 2020 Jan 29;18(4):802–14. doi: 10.1111/jth.14725 (PMC7186824; doi:10.1111/jth.14725)
Supplement: Supplementary file 1 [file JTH-18-802-s001.pdf]

Fibrinogen αC-regions are not directly involved in fibrin polymerization as evidenced by a "Double-Detroit" recombinant fibrinogen mutant and knobs-mimic peptides

Cédric Duval, Aldo Profumo, Anna Aprile, Annalisa Salis, Enrico Millo, Gianluca Damonte, Julia S. Gauer, Robert A.S. Ariëns, and Mattia Rocco

SUPPORTING INFORMATION

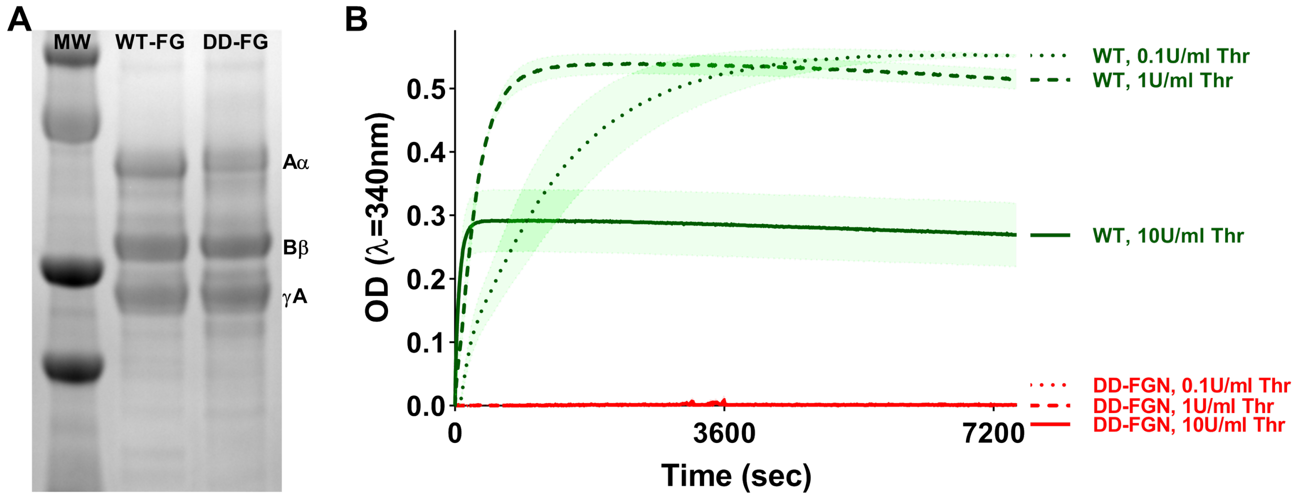

Supporting Figure 1: Production and characterization of WT-FG and DD-FG. Panel A: SDS-PAGE of both recombinant WT-FG and DD-FG, showing the Aα, Bβ and γA chains, and the absence of contaminants. Panel B: turbidity analysis using a range of thrombin concentrations from low (0.1 IU/mL) to high (10 IU/mL), showing that DD-FG does not exhibit an increase in optical density (OD), as observed for WT-FG.

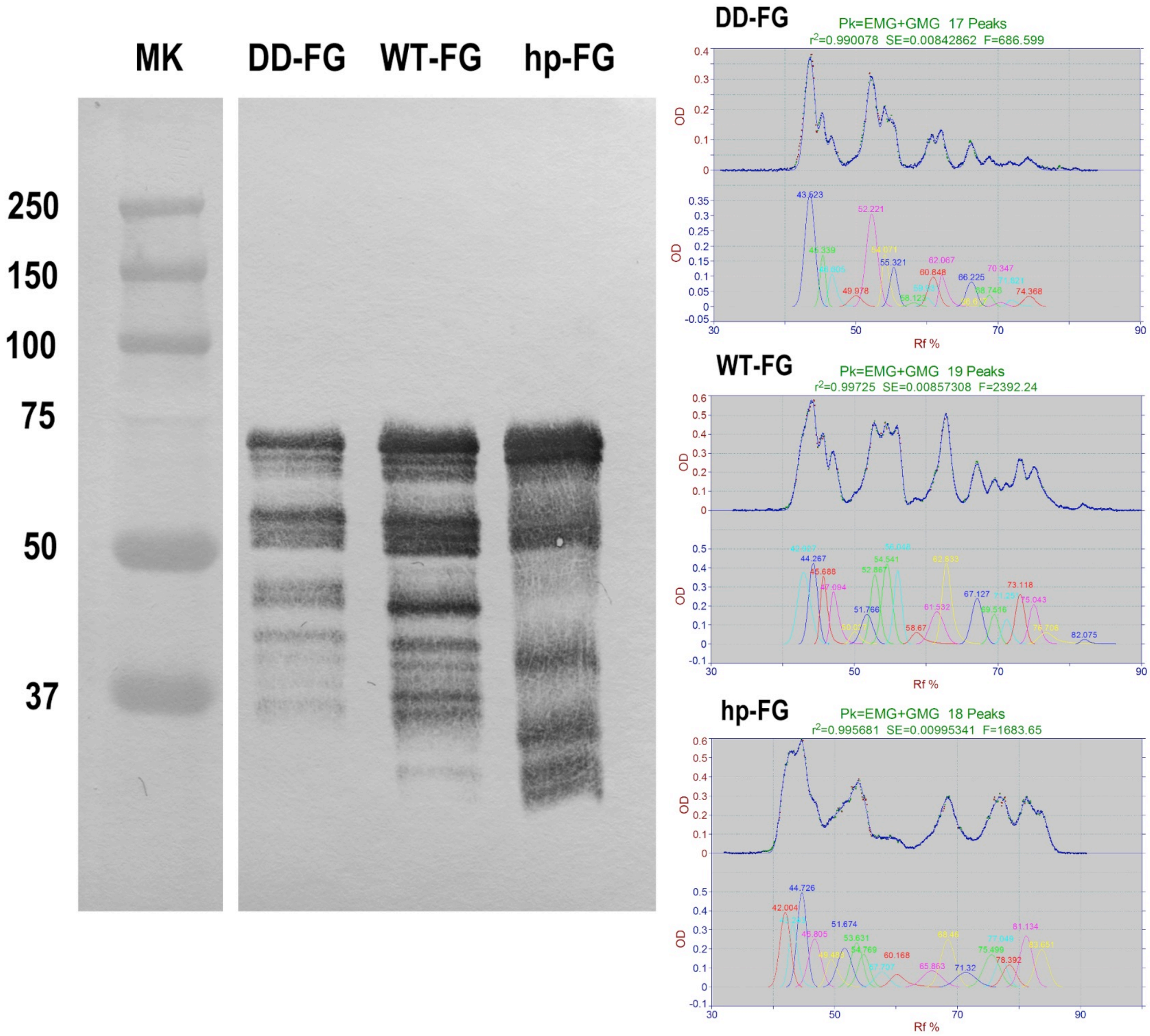

Supporting Figure 2: Aα-chains integrity controls. Left panel, SDS-PAGE followed by Western-blot analysis with HRP-staining using the Y18 monoclonal antibody to the N-terminus of the Aα-chain for DD-FG, WT-FG, and hp-FG. A broad molecular weight (MW) marker (MK) was included in the gel/blot, with the approximate MW values indicated on the left side. The three panels on the right side show the results of densitometric analyses (upper traces) followed by PeakFit deconvolution (lower traces) for, from the top, DD-FG, WT-FG, and hp-FG. A log(MW) vs. % relative migration distance (Rf) calibration line was built using the three lower MW points, and was then employed to deduce the approximate MW of the samples' deconvoluted peaks.

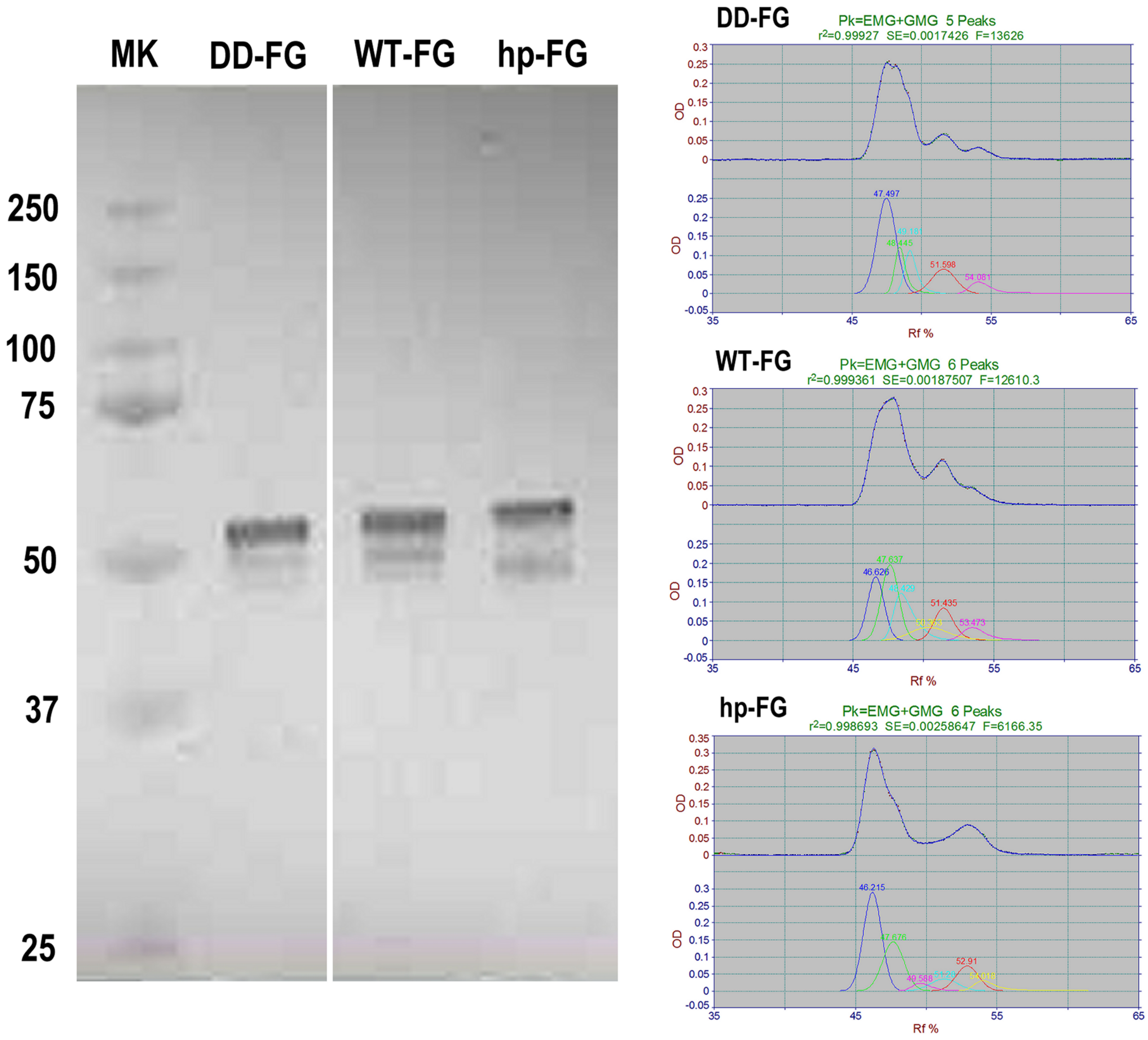

Supporting Figure 3: Bβ-chains integrity controls. Left panel, SDS-PAGE followed by Western-blot analysis with HRP-staining using a polyclonal antibody to the C-terminal part of the Bβ-chain for DD-FG, WT-FG, and hp-FG. A broad molecular weight (MW) marker (MK) was included in the gel/blot, with the approximate MW values indicated on the left side. The three panels on the right side show the results of densitometric analyses (upper traces) followed by PeakFit deconvolution (lower traces) for, from the top, DD-FG, WT-FG, and hp-FG. A log(MW) vs. % relative migration distance (Rf) calibration line was built using the three lower MW points, and was then employed to deduce the approximate MW of the samples' deconvoluted peaks.

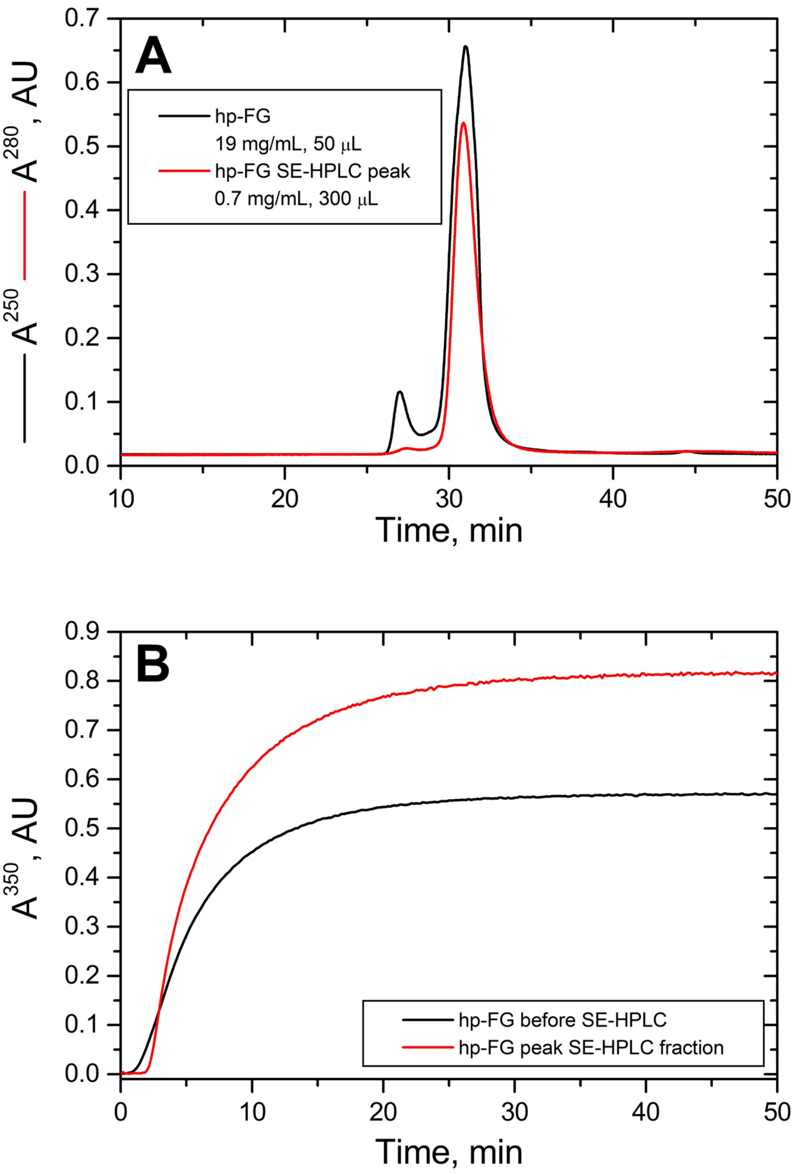

Supporting Figure 4: Influence of aggregates on the turbidity profiles of fibrinogen following activation with thrombin. Panel A, SE-HPLC traces in semi-preparative (black trace, absorbance monitored at 250 nm) and analytical (red trace, absorbance monitored at 280 nm) modes of hp-FG samples. In the semi-preparative mode, 40 μL of hpFG at 19 mg/mL were injected in the SE-HPLC system and eluted with TBS at 0.4 mL/min (black trace). The monomer peak fraction (from ~29.8 to~30.8 min) was manually collected, resulting in ~0.4 mL at 0.7 mg/mL, of which 300 μL were re-injected in the SE-HPLC system (red trace) and the remaining was used for the turbidity experiment. Panel B, absorbance at 350 nm profiles of hp-FG at 0.3 mg/mL before (black trace) and after (red trace) removal of high molecular weight species. Samples were treated with thrombin at final 0.08 NHIu/mL (0.28 NIHu/mg FG) using the same protocol as for the turbidity experiments described in the main text.

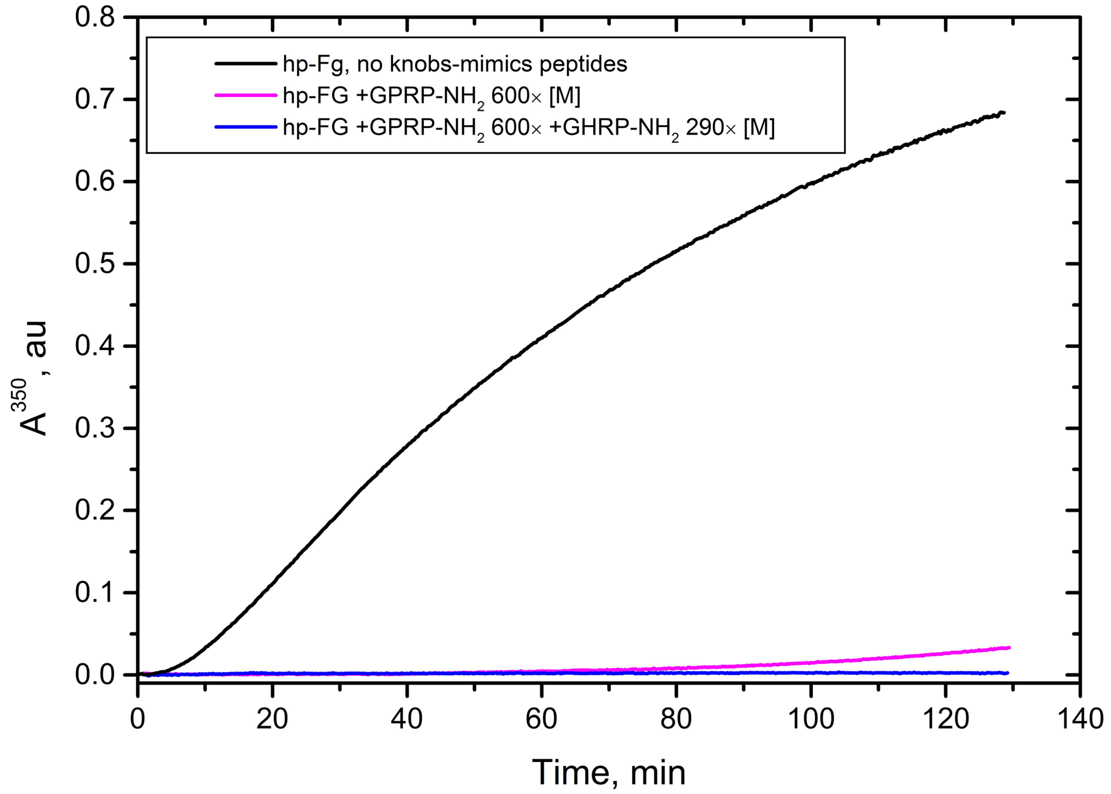

Supporting Figure 5: Effects of GPRP-NH_2 and GHRP-NH_2 peptide knobs mimics on the turbidity profile of hp-FG following thrombin activation. Hp-FG solutions at 1.2 mg/mL in TBS without knobs-mimic peptides (black trace), in the presence of GPRP-NH_2 alone at 2.23 mM (600× [M]; magenta trace), and in the presence of both GPRP-NH_2 2.23 mM and GHRP-NH_2 1.06 mM (290× [M]; blue trace) were treated with thrombin at final 0.12 NIHu/mL (0.1 NIHu/mg FG).

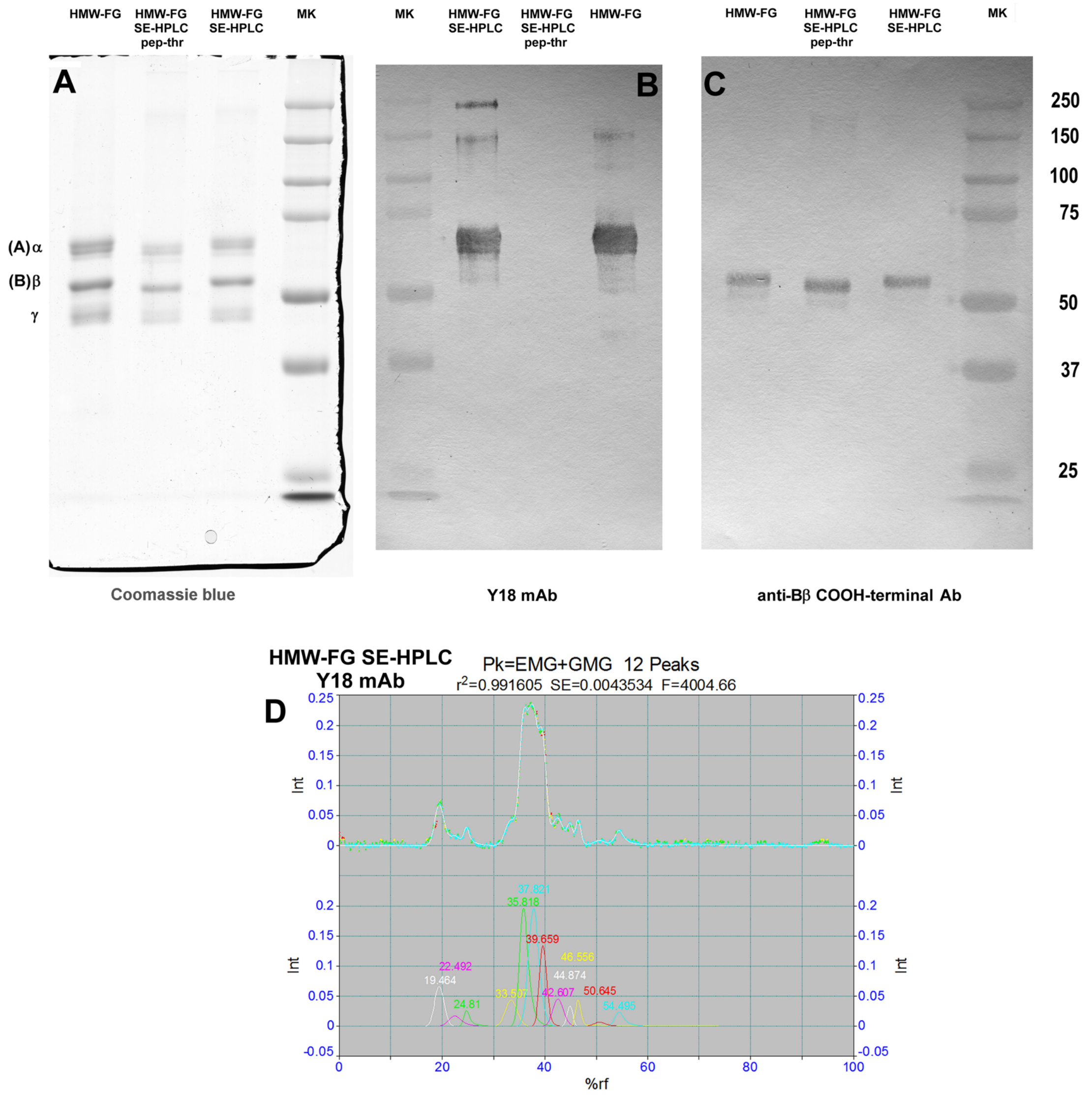

Supporting Figure 6: Quality control of HMW-FG samples. Panel A, SDS-PAGE with Coomassie-blue staining of the starting stock (first lane), of the SE-HPLC purified material (third lane), and of the SLS/DLS sample with GPRP-NH_2 and GHRP-NH_2 500× [M] after 1h incubation with 2 NIHu/mg FG thrombin (second lane); the fourth lane contains the molecular weight (MW) markers (MK). Panel B, same samples as in panel A after Western-blotting and HRP-staining with the Y18 mAb against the N-terminus of the Aα-chain; the marker is in the first lane, followed by the HMW-FG samples in reverse order in respect to panel A (note the complete absence of staining in the third lane, indicating complete removal of FpA). Panel C, same samples and order as in panel A, after Western-blotting and HRP-staining with a polyclonal Ab against the COOH-terminal of the (B)β chain. Panel D, results of the densitometric analysis (upper traces) followed by PeakFit deconvolution (lower traces) for the SE-HPLC-purified HMW-FG, Y18 HRP-stained sample (panel B, fourth lane). The approximate locations of the FG chains are indicated on the left side, and the indicative MW of the markers are reported on the right side (top panels).

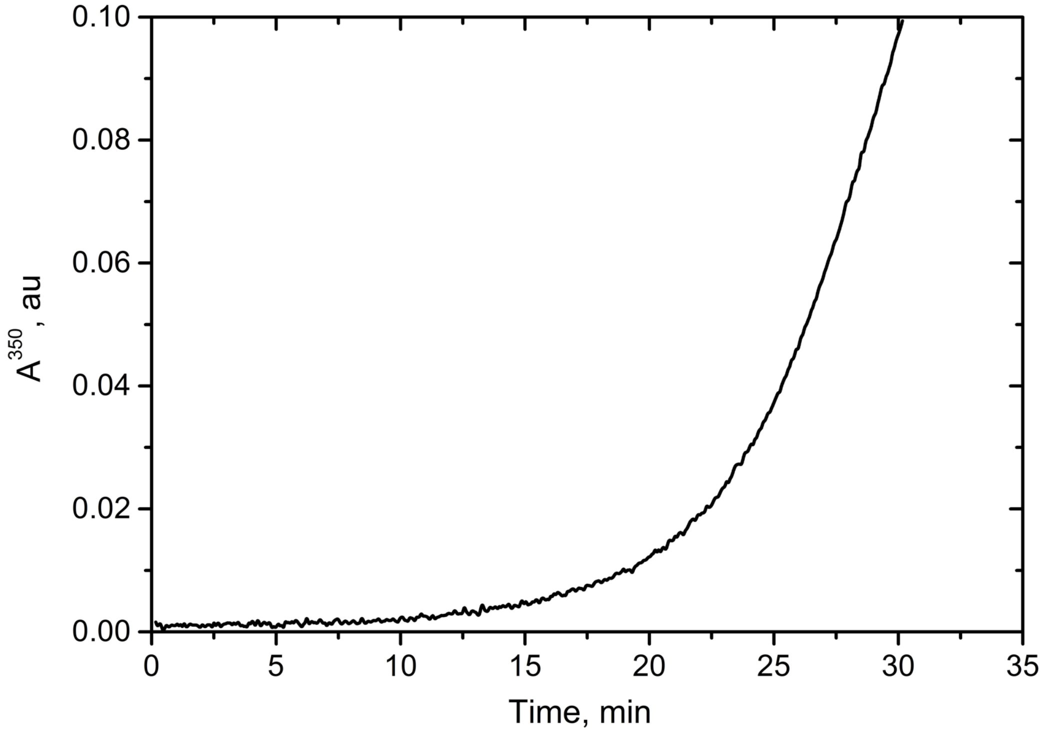

Supporting Fig. 7: Turbidity profile recorded on the hp-FG used for the SLS/DLS experiment of Fig. 4. SE-HPLC-purified hp-FG at 0.21 mg/mL activated with thrombin at a final concentration of 0.032 NIHu/mL (0.15 NIHu/mg FG) in TBS. Only the first 30 minutes of the reaction are shown, highlighting that the experiments reported in Fig 4 of the main text concern data taken mainly during the lag-time of fibrin polymerization.

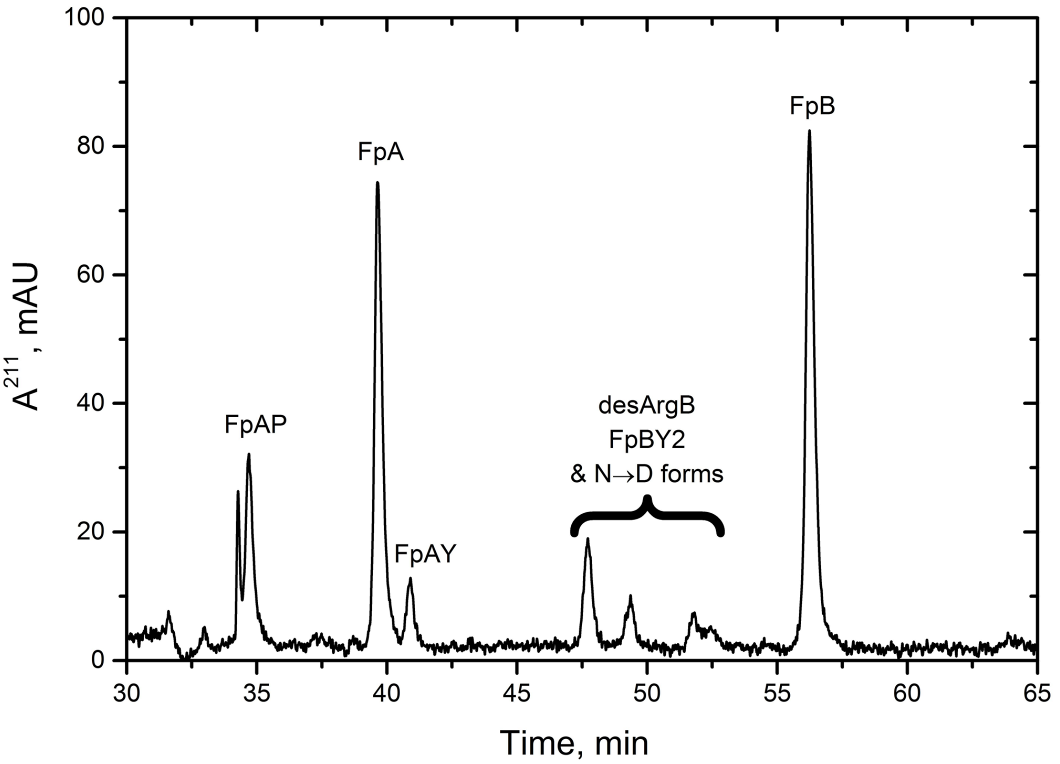

Supporting Figure 8: Fibrinopeptides release for HMW-FG + knobs-mimic peptides after 1 h thrombin treatment in the SLS/DLS cuvette. After the SLS/DLS experiment (see Fig. 4B), 40 μL of the HMW-FG solution at 0.8 mg/mL containing GPRP-NH_2 and GHRP-NH_2 both 500× [M] and thrombin at 1.54 NIHu/mL, and 40 μL of the corresponding TBS buffer with the same amount of peptides and thrombin, were boiled for 2 min and then filtered and RP-HPLC analyzed (20 μL injections) as reported in ***Fibrinopeptides release analysis*** (see below). No marker was added to this sample. The graph reports the A^{211} of the sample after pointwise subtraction of the A^{211} of the TBS sample.

Supporting Table 1: Mutagenesis primers sequences.

	**Forward Primer**	**Reverse Primer**
**Aα-R16S**	5'-ggaggaggcgtg**agt**ggcccaaggg-3'	5'-cccttgggcc**act**cacgcctcctcc-3'
**Bβ-R14S**	5'-ggtttcttcagtgcc**agt**ggtcatcgacccc-3'	5'-ggggtcgatgaccactggc**act**gaagaaacc-3'

Supporting Table 2: Densitometric analysis of a Western-blot HRP-stained with the Y18 mAb against the N-terminus of the Aα-chain.

Hp-FG			WT-FG			DD-FG		
**Rf%**	**Mol. wt.**	**Area %**	**Rf%**	**Mol. wt.**	**Area %**	**Rf%**	**Mol. wt.**	**Area %**
42.0	70,188	9.00						
43.2	68,409	6.35	42.9	68,858	10.62	43.5	68,013	23.29
44.7	66,339	10.30	44.3	66,972	8.22			
			45.7	65,030	5.54	45.3	65,501	5.86
46.8	63,541	6.96	47.1	63,162	5.25	46.6	63,805	5.40
Group 1 sum (%)		32.62	Group 1 sum (%)		29.63	Group 1 sum (%)		34.55
49.5	60,105	3.87	50.0	59,437	1.49	50.0	59,498	2.88
51.7	57,442	6.39	51.8	57,333	4.02	52.2	56,795	21.36
			52.9	56,040	7.42			
53.6	55,159	6.05	54.5	54,130	9.35	54.1	54,659	7.89
54.8	53,874	4.44	56.0	52,465	6.61	55.3	53,261	6.36
57.7	50,692	2.53	58.7	49,690	1.91	58.1	50,256	0.90
						59.9	48,408	1.62
60.2	48,171	2.42	61.5	46,829	5.19	60.8	47,497	6.47
			62.8	45,583	9.39	62.1	46,313	5.31
Group 2 sum (%)		25.70	Group 2 sum (%)		45.38	Group 2 sum (%)		52.79
						66.2	42,489	5.29
65.9	42,809	3.65	67.1	41,702	5.89	66.6	42,145	0.34
68.5	40,565	7.83	69.5	39,688	3.10	68.7	40,326	1.87
71.3	38,231	3.37	71.3	38,286	2.71	70.3	39,010	1.02
						71.8	37,837	1.45
Group 3 sum (%)		14.85	Group 3 sum (%)		11.70	Group 3 sum (%)		9.97
			73.1	36,833	5.33			
75.5	35,060	6.08	75.0	35,392	4.79	74.4	35,891	2.69
77.0	33,951	4.04	76.7	34,194	2.49			
78.4	33,019	3.49						
81.1	31,196	7.46	82.1	30,593	0.69			
83.7	29,610	5.76						
Group 4 sum (%)		26.83	Group 4 sum (%)		13.29	Group 4 sum (%)		2.69

Supporting Table 3: Densitometric analysis of a Western-blot HRP-stained with the anti Bβ-chain C-terminal region polyclonal antibody.

hp-FG			WT-FG			DD-FG		
**Rf%**	**Mol. Wt.**	**Area %**	**Rf%**	**Mol. wt.**	**Area %**	**Rf%**	**Mol. wt.**	**Area %**
46.2	54,108	42.98	46.6	53,766	21.48			
47.7	52,902	25.62	47.6	52,934	27.00	47.5	53,048	46.54
			48.4	52,291	20.13	48.4	52,278	13.47
49.6	51,364	3.59	50.4	50,754	10.99	49.2	51,688	13.83
Intact Bβ sum (%)		72.19	Intact Bβ sum (%)		68.62	Intact Bβ sum (%)		73.85
51.3	50,041	7.97	51.4	49,921	13.45	51.6	49,796	15.21
52.9	48,798	13.61	53.5	48,376	6.94			
54.0	47,971	6.23				54.1	47,924	10.94
Degraded Bβ sum (%)		27.81	Degraded Bβ sum (%)		31.38	Degraded Bβ sum (%)		26.15

Supporting Table 4: Predicted $[\langle M^{*}\rangle_{w}]_{wa}$ that would be measured by SLS of HMW-FG species in the presence of GPRP-NH_2 and GHRP-NH_2 500× [M] after thrombin treatment, as a function of discrete weight fractions (w_D) of dimers, followed by statistical evaluation against measured data[a].

	Measured $[\langle M^{*}\rangle_{w}]_{wa}$ (g/mol)<br>41 to 60 min after thrombin addition	N 20 s acquisitions<br>prior to thrombin	Equivalent<br>sample size $\hat{n}'_e$	N 10 s acquisitions<br>with thrombin	Equivalent<br>sample size $\hat{m}'_e$
	323,000 ± 7,000	27	14	95	51
w_D	Computed $(\langle M\rangle_{w})_{D}$ (g/mol)	Pooled SD (g/mol)	t	p < 0.05 (DF = 63)	p < 0.05 (DF = 2)
0.00	318,000 ± 2,500[b]	na[c]	na	na	na
0.03	327,000 ± 3,000	6,600	-4.3755	0.00002	0.02424
0.05	334,000 ± 3,000	6,600	-11.8659	< 0.00001	0.00352
0.10	349,000 ± 3,000	6,600	-27.9167	< 0.00001	0.00064
0.13	359,000 ± 3,000	6,600	-38.6172	< 0.00001	0.00034
0.15	365,000 ± 3,000	6,600	-45.0375	< 0.00001	0.00027
1.00	635,000 ± 5,000[d]	na	na	na	na

[a]The one-tailed Student's *t*-test *p*-values at the 95% confidence level were computed on-line from the calculated *t*-values and DF (https://www.socscistatistics.com/pvalues/tdistribution.aspx)

[b]Experimental $[\langle M^{0}\rangle_{w}]_{wa}$ value prior to thrombin addition minus the calculated mol. wt. of two FpA and two FpB.

[c]na: not applicable

[d]Computed HMW-FG dimer $[\langle M^{0}\rangle_{w}]_{wa}$ using the monomer value prior to thrombin addition minus the calculated mol. wt. of two FpA and two FpB.

Supporting Table 5: Predicted [<M*>$_w$]$_{wa}$ that would be measured by SLS of DD-FG species after thrombin treatment, as a function of discrete weight fractions (w_D) of dimers, followed by statistical evaluation against measured data[a]

	Measured [<M*>$_w$]$_{wa}$ (g/mol) 45 to 52 min after thrombin addition	N 20 s acquisitions prior to thrombin	Equivalent sample size $\hat{n}'_e$	N 1 s acquisitions with thrombin	Equivalent sample size $\hat{m}'_e$
	322,000 ± 36,000	47	7	323	50
w_D	Computed (<M>$_w$)$_D$ (g/mol)	Pooled SD (g/mol)	t	p < 0.05 (DF = 55)	p < 0.05 (DF = 2)
0.00	322,000 ± 13,000[b]	na[c]	na	na	na
0.03	331,000 ± 14,000	33,700	-1.9286	0.02951	0.09683
0.05	338,000 ± 14,000	33,700	-3.3991	0.00063	0.03836
0.10	354,000 ± 14,000	33,700	-6.7604	< 0.00001	0.01060
0.13	363,000 ± 15,000	33,700	-8.6373	< 0.00001	0.00657
0.15	370,000 ± 15,000	33,700	-10.1055	< 0.00001	0.00483
1.00	643,000 ± 26,000[d]	na	na	na	na

[a]The one-tailed Student's t-test p-values at the 95% confidence level were computed on-line from the calculated t-values and DF (https://www.socscistatistics.com/pvalues/tdistribution.aspx)

[b]Experimental [<M^0>$_w$]$_{wa}$ value prior to thrombin addition minus the calculated mol. wt. of two FpA and two FpB.

[c]na: not applicable

[d]Computed DD-FG dimer [<M^0>$_w$]$_{wa}$ using the monomer experimental value prior to thrombin addition minus the calculated mol. wt. of two FpA and two FpB.

Supporting Information for Materials and Methods sections

DD-FG and WT-FG expression and purification

Recombinant human AαR19S and BβR17S FG (DD-FG) were prepared by inserting cDNA from FGA and FGB clones into pSELECT-Zeo and pSELECT-Puro plasmids (Invivogen, Toulouse, France), respectively. Both FGA and FGB containing pSELECT plasmids were subjected to site-directed mutagenesis where residues FGA-19R and FGB-17R were mutated to serine (S) (primer sequences in Supplementary Table 1), using a QuickChange site-directed mutagenesis kit (Agilent Technologies, Stockport, UK). Chinese hamster ovary (CHO) cells, already containing the human γA chain (using pMLP-γ plasmid and clone selection by resistance to geneticin) were co-transfected with the mutated pSELECT-Zeo/FGA-R19S and pSELECT-Puro/FGB-R17S vectors. Clones expressing the mutant fibrinogen were selected by resistance to zeocin and puromycin (Invivogen) and expression levels were monitored using an in-house ELISA assay (see below). CHO cells expressing either human wildtype fibrinogen (WT-FG) or DD-FG were grown in Petri dishes, and transferred into roller bottles containing adherent microcarrier beads and DMEM F12 medium (ThermoFisher Scientific, Altrincham, UK) supplemented with 2 mg/mL aprotinin, 5 mg/mL insulin, and transferrin sodium selenite supplement (Roche, Burgess Hill, UK). The medium was harvested and replaced every 48 h, and stored at -80 °C in the presence of 150 μM PMSF. Medium was harvested for as long as fibrinogen was detectable by ELISA. Fibrinogen was precipitated overnight with 40% saturated ammonium sulphate (ThermoFisher Scientific) and purified by calcium-dependent IF-1 mAb (Kamiya Biomedical, Seattle, WA, USA) affinity chromatography, as previously described [1]. Prior to chromatography, $CaCl_2$ was added to the samples (10 mM final). The fibrinogens were eluted in Tris Buffered Saline [TBS: tris(hydroxymethyl)aminomethane 50 mM, NaCl 100mM, pH7.4] containing 5 mM EDTA, and dialyzed overnight against TBS.

Fibrinogen ELISA assay

Maxisorp Nunc-Immuno MicroWell 96-well plates (ThermoFisher Scientific) were coated with 100 μL of DAKO polyclonal rabbit anti-human fibrinogen antibody (Agilent) diluted 1:4000 in coating buffer (100 mM Na_2CO_3, pH 9.6) for 1 h, and the plates were then blocked overnight at 4 °C with 300 μL of 3% bovine serum albumin (BSA) in dilution buffer (10 mM Na_2HPO_4, 1.8 mM KH_2PO_4, 2.7 mM KCl, 137 mM NaCl, 1% BSA, 0.05% Tween20, pH 7.4). Plates were washed with washing buffer (10 mM Na_2HPO_4, 1.8 mM KH_2PO_4,

2.7 mM KCl, 500 mM NaCl, 0.05 % Tween20, pH 7.4), before 100 μL samples and standard (0, 15 to 1000 ng/mL) were added to the wells and incubated for 2 h. Following washes, plates were incubated with 100 μL polyclonal Sheep anti-Human fibrinogen-HRP antibody (Enzyme Research Laboratories (ERL), Swansea, UK) diluted 1:1000 in dilution buffer for 1 h. Following washes, 100 μL of 0.5 mg/mL 1,2-Phenylenediamine Dihydrochloride (OPD; diluted in 50 mM Na_2HPO_4, 25 mM citric acid, pH 5.0; supplemented with 1.2 % H_2O_2) was added for 5 min before the reaction was stopped with 200 μL of 3 M H_2SO_4. Absorbency was read at 490 nm using a PowerWave HT Microplate Spectrophotometer (BioTek, Swindon, UK), and the sample concentrations were calculated using the standard curve.

Thrombin reconstitution and activity determination

Each time a thrombin vial was thawed, the activity was determined using a modified Biggs method [2], as previously described [3] but with some additional changes. In short, 200 μL of a 1.1 mg/mL human plasma fibrinogen (FIB3 from ERL) solution in 5.45 mM Na-citrate, 0.425% (w/v) HEPES (N-2-hydroxyethylpiperazine-N'-2-ethanesulfonic acid), pH 7.4, were carefully pipetted into five plastic UV-transparent, 10 mm path length micro cuvettes (Plastibrand; Sigma-Aldrich), which were placed in the thermostatted 6-position sample changer of a Beckman DU-640 spectrophotometer. The temperature, 25.0 ± 0.1 °C (instead of 37 °C as previously done [3]), maintained by a recirculating water bath, was controlled with a small flexible, plastic covered thermistor probe immersed in water inside a stoppered quartz cuvette placed in the 6th position of the sample changer and monitored with a 08403 series digital thermometer (Cole-Parmer, Vernon Hill, IL, USA). Cuvettes were matched at the analytical wavelength of 350 nm, and 100 μL of 0.85% HEPES, pH 7.4 were added to the first cuvette used as a blank. A kinetics method lasting 30 min with a 10 s reading interval for the five cuvettes (read time 0.7 s for each cuvette) was programmed.

The thrombin calibration curve was prepared once by accurately dissolving the content of a WHO International Standard ampoule (WHO 2nd International Standard for Thrombin 01/580 US, FDA/CBER Thrombin Standard Lot K, nominal 110 IU, code 01/580; NIBSC, Potters Bar, UK; http://www.nibsc.org/) with 0.993 mg of MQ water (weighed on a precision balance). Immediately, 20 μL were diluted with 980 μL of 0.85% HEPES, pH 7.4, obtaining a 2.2 IU/mL (corresponding to 1.91 NIHu/mL [4]) stock solution. Four 0.5 mL dilutions in the same buffer were then immediately made, at 0.075, 0.060, 0.045 and 0.030 IU/mL. From each dilution, 100 μL were quickly pipetted with remixing at 15 s intervals into each of the other four cuvettes (a timer was started at the first injection), and at the 1 min

mark the data acquisition was launched. Blank-corrected, and starting time adjusted data (to 400 s) were analyzed with TableCurve 2D (SigmaPlot, now distributed by Systat Software, San José, CA, USA) using the Cascade transition function, $y = a + b\,\{1 + [c \exp(-d\,(x - e)) - d \exp(-c\,(x - e))] / (d - c)\}$, with y being the corrected A^{350}, and x being the adjusted time. The clotting time (*Ct*) was taken at the beginning of the rise of the A^{350}, corresponding to the "*e*" term in the Cascade function. By plotting 1/*Ct* vs. the nominal NIHu/mL in each cuvette, a calibration curve was obtained. It was fitted with a straight line, with slope 0.2403 s^{-1} $NIHu^{-1}$ mL and intercept 0.0001 s^{-1}. For the determination of working thrombin solutions, each FG-containing cuvette except the blank received the same thrombin dilution, targeted to be ~0.04 NIHu/mL, and the *Ct*s were averaged before the calculation of the activity.

Turbidity coupled to fibrinopeptides release

For each sample, 150 μL of reacting solution were prepared by diluting a fibrinogen stock to 0.6 mg/mL in TBS buffer having a final polyethylene glycol (PEG) 8000 concentration of 0.1% (TBS-PEG), and sufficient HPLC marker (MK; see below). From this mixture, 110 μL were pipetted in the plastic micro UV-transparent, 10 mm path length cuvettes, that were placed in the Beckman DU-640 spectrophotometer 6-cuvettes changer, thermostated at 25.0 ± 0.1 °C, in this order: hp-FG (blank), DD-FG, WT-FG, hp-FG. The wavelength was set at 350 nm, and the cuvettes were matched to offset their differences. The activating solution was prepared by diluting a thrombin stock solution, the activity of which was previously titrated (see above), to 0.169 NIHu/mL in TBS-PEG. When mixed 1:1 with the fibrinogen preparations, thrombin activity was 0.084 NIHu/mL and fibrinogen concentration 0.3 mg/mL (0.28 NIHu/mg FG).

In a thermostatting block (Thermomixer Comfort; Eppendorf, Hamburg, Germany) maintained at 25 °C, three 0.5 mL empty Eppendorf tubes were prepared for each reaction mixture. To begin with, 110 μL of TBS-PEG were pipetted in the first (blank) cuvette containing hp-FG. The activation was carried out directly in the other cuvettes positioned in the spectrophotometer, by pipetting and mixing at 30 s intervals 110 μL of the thrombin solution, and then taking out 110 μL of the activated mixture. The spectrophotometer data acquisition was started 10 s after the last pipetting. Each retrieved 110 μL was transferred to one of the 0.5 μL Eppendorf tubes, from which rapidly 2 x 30 μL were taken and deposited in the other two tubes. After 1 h, 2 h, and 3 h, the corresponding reactions were stopped by boiling, cooling, and filtration (see ***Fibrinopeptides release analysis*** below). The turbidity was monitored at 10 s intervals (for all 4 cuvettes used; read time 0.7 s for each cuvette) for almost 2 h, and then every 30 s for up to 4 h.

At the end of the experiment, an electrophoresis sample was prepared for the activated hp-FG and WT-FG by adding the required amount of SDS/DTT reducing buffer [5] directly in the turbidity cuvette, and retrieving it after several hours of incubation with gentle shaking to ensure clot dissolution, followed by 5 min boiling. Reduced SDS-PAGE samples for all fibrinogens before activation, and for the activated DD-FG sample that did not clot, were prepared without the long incubation times.

Fibrinopeptides release analysis

Preliminary fibrinopeptide release analyses were done at both low and high thrombin concentrations, using procedures similar to those described above, but without the spectrophotometer part. Fib1 hp-FG was from ERL, prepared as described in the ***Thrombin reconstitution and activity determination*** section, except that the dialysis step was against TBS, and that aliquots of 0.5 ml at ~22 mg/mL were made and stored at -80 °C.

An internal standard marker (MK) was introduced to make the quantification of the Fps more reliable. After several trials (unpublished thesis work of D. Pacini, 2007-08 and A. Tucci, 2013-14; University of Genova, Italy), the synthetic peptide Acetyl-HN-GLKGEF-COOH was found to give the best results, eluting well-separated between the FpAP and FpA peaks. It was manually synthesized as described below (***Peptide synthesis and stock solutions preparation***). This marker was found not to interfere with thrombin activity and did not show degradation effects over the Fps release assays incubation times (unpublished thesis work cited above), and was therefore spiked in all reaction mixtures destined to Fps analysis in a quantity so to give an UV signal at 211 nm comparable with that of FpA at plateau level.

Reactions were carried out at 25 °C in the thermostatted block for 90 or 120 min with FGs at either 0.8 or 0.42 mg/mL, activated with thrombin at 0.5 (low) or 2.84 (high) NIHu/mL. The reactions were stopped by putting the tubes in boiling water for 1 min, cooled in ice water, and the content first spun for 10 min at 19,000 *g* and then vacuum filtered on a 96-well plate with 0.45 μm pore-size hydrophilic, low-protein binding Durapore membrane (MSHVN4510; Merck-Millipore, Cork, Ireland) in a FreedomEvo liquid handling system (TECAN, Männedorf, Switzerland) prior to loading in the HPLC autosampler. Fps were analyzed by RP-HPLC on a 1.0 × 150 mm, 300 Å pore/size, 3.5 μm particle size Symmetry 300 C18 column, protected by a Symmetry 300 C18 2.1 × 10 mm pre-column (Waters, Milford, MA, USA) as already reported [6]. The HPLC set-up consisted of a 1200 series system (Agilent), equipped with G1379B degasser, G1376A capillary pump, G1377A micro autosampler, G1316A thermostated column holder, and G1315B diode

array detector (DAD) with a 0.5 μL flow cell (G1315-68724, 10.00 ± 0.02 mm path length). It was equilibrated in 97% 25 mM CH_3COONH_4, brought to pH 6 with diluted H_3PO_4 (buffer A) and 3% 50 mM CH_3COONH_4, brought to pH 6 with diluted H_3PO_4, and further diluted 1:1 with CH_3CN (buffer B). The flow rate was 50 μl/min, and the elution was performed in this sequence: isocratic 97% A - 3% B for 8 min, then three linear gradients, the first in 5 min to 12% B, the second in 45 min to 27% B, and the third in 6 min to 100% B, and finally maintained at 100% B for 5 min. The elution was monitored at 211 nm, and the column was temperature-controlled at 30 °C. Areas (mAU × min) under the peaks were determined using PeakFit v4 (Jandel Scientific, now distributed by Systat Software, Richmond, CA), after subtracting the absorbance of a blank run, where only the reaction buffer plus thrombin was injected after boiling/filtering. The exponentially modified Gaussian plus Gaussian modified Gaussian (EMG + GMG) function was used. To determine the correct ratio between total FpA and FpB amounts, molar concentrations of fibrinopeptide species in each chromatographic peak were determined from calculated molar extinction coefficients at 211 nm (ε^{211}, M^{-1} cm^{-1}). First, the theoretical ε at 205 nm (ε^{205}) for FpA and FpB (40,800 and 49,300 M^{-1} cm^{-1}, respectively) were taken from Higgins and Shafer [7], with appropriate corrections for species missing one residue. Then, each ε^{205} was converted to ε^{210} by dividing it by a weighted average ratio of $\varepsilon^{205}/\varepsilon^{210}$ (1.561) deduced from the data in Table 1 of Goldfarb *et al.* [8] for a set of proteins. Finally, the spectra recorded by the HPLC DAD at the top of the FpA and FpB peaks were analyzed to recover their A^{210}/A^{211} ratio (1.077 for FpA; 1.064 for FpB) and the calculated ε^{210} were thus converted to ε^{211}, 211 nm being the wavelength at which our HPLC analyses were monitored for historical reasons (normalization of triplicate samples in fibrinopeptide release analyses [9]), since they are less noisy than at 210 nm. Absolute amounts were calculated from the areas by first multiplying them by the flow rate and then dividing them by the appropriate calculated molar extinction coefficient.

Peptide synthesis and stock solutions preparation

The marker peptide acetyl-HN-GLKGEF-COOH and the *B*-knob-mimic GHRP-NH_2 were synthesized using the standard method of solid phase peptide synthesis following the 9-fluorenylmethoxycarbonyl (Fmoc) strategy with minor modifications [10,11]. Reagents and solvents were purchased from Sigma-Aldrich, Alfa Aesar (Thermo Fisher, Karlsruhe, Germany), Biosolve BV (Valkenswaard, The Netherlands) and VWR International (Milano, Italy). The marker peptide sequence was chemically modified by the introduction of an acetyl group on the solid support to the amino terminal of the protected peptide sequence. The peptides were then purified by preparative reverse-phase HPLC (RP-HPLC) on a

1260 Infinity HPLC (Agilent) equipped with a 21.2 × 250 mm C18 Luna column (Phenomenex, Torrance, CA, USA), followed by lyophilization. The identity and purity of the peptides were verified by mass spectroscopy analysis as described below (***Mass spectroscopy***).

For the marker peptide, a stock solution was prepared in MQ water at ~100× fold concentration in respect to that used in the Fps release assays working solutions. Commercial GPRP-NH_2 and synthesized GHRP-NH_2 were dissolved in TBS at nominal concentrations by weight of 74 and 86 mM, respectively, but the latter presented some undissolved material. Their effective concentrations were then determined by diluting the stocks 1:1000 in MQ water and measuring their absorbance at 205 nm against a 1:1000 dilution of TBS in MQ water (5 scans were averaged). Molar extinction coefficients at 205 nm were computed on-line from the peptides sequence (Protein Parameter Calculator; http://nickanthis.com/tools/a205.html) [12], resulting in ε^{205} = 9,690 M^{-1} cm^{-1} for GPRP-NH_2 and ε^{205} = 14,890 M^{-1} cm^{-1} for GHRP-NH_2, whose stock solutions where then ascertained to be 89 and 51 mM, respectively.

Mass spectroscopy

LC-MS analyses were carried out in an 1100 HPLC-MSD Ion Trap XCT system (Agilent), equipped with an electrospray ion source (HPLC-ESI–MS) (Agilent). Separations were performed as reported above (***Fibrinopeptides analysis***). Ions were detected in ion charged control with a target ion value of 70,000 and an accumulation time of 300 ms, using a capillary voltage of 3300 V and a nebulizer pressure of 15 psi. Other conditions were: drying gas, 8 L/min; dry temperature, 325 °C; rolling averages, 2; averages, 5. Mass spectra were acquired in the positive ion mode in a range of 300–1800 m/z consistent with expected mass charge ratios. MS^2 analysis was conducted using fragmentation amplitude at 1 V and the MS^2 spectra of the three most abundant ions were collected if it exceeded a threshold Abs of 10000. Raw MS files were processed with Data Analysis for LC/MSD Trap version 3.3 (Bruker Daltonik GmbH, Macerata, Italy).

Size-exclusion chromatography

A HPLC series 200 system (Perkin-Elmer, Milano, Italy), comprising vacuum solvent degasser, pump, and UV-Vis monitor, and a manual Altex 210 A injector valve with a 500 μL PEEK loop were utilized. SE-HPLC was performed on two TSK 300 × 7.8 mm columns in series (G4000PW_{XL} and G3000PW_{XL}; Tosoh Bioscience, Tokyo, Japan), protected by a similarly packed 6 × 40 mm guard column. The flow rate was 0.4 mL/min, TBS the solvent, and the absorbance was monitored at either 250 (high concentration

samples) or 280 nm. Some samples were concentrated using Amicon Ultra 0.5 mL, 50K cut-off Centrifugal Filters (Sigma-Aldrich), following the manufacturer's instructions. Prior to injection, samples were spin-filtered using 0.22 μm pore-size Spin-X filters (Sigma-Aldrich). In the semi-preparative mode, a single fraction beginning and ending at approximately half of the absorbance of the main peak was manually collected.

Static and Dynamic light scattering theory and working conditions

Light-scattering (LS) is an analytical technique that can be performed in two main modes, "static" LS (SLS), and "dynamic" LS (DLS) [13]. In the first mode, the intensity of the scattered light at one (usually 90°) or several scattering angles (multi-angle LS, MALS) is collected for some time and averaged. It is directly proportional to the weight-average molecular weight $<M>_w$, and for elongated particles in MALS mode can also yield the *z*-average radius of gyration $<R_g>_z$, of all the species present in solution. In the DLS mode, the fluctuations of the instantaneous intensity of the scattered light, usually collected at the 90° scattering angle, are processed by an autocorrelator, and from the fit of the autocorrelation function the *z*-average translational diffusion coefficient $<D_t>_z$ of all species in solution is obtained. $<D_t>_z$ is usually converted into the hydrodynamic (or Stokes') radius $<R_s>_z$ using the Stokes-Einstein equation [13]. SLS is indicated for time-resolved studies because collection intervals of 0.5÷1 s are usually more than sufficient for good averages. Instead, processing of the DLS signal normally requires longer collection times, especially for large particles. However, the intensity signal is also recorded (as counts/seconds), so a DLS instrument can also provide a "static" signal.

Data were collected with either 1, 10, or 20 s acquisition times, at a precisely controlled T of 20.0 ± 0.0 °C. The 1 s acquisition times were always used during the early phases following fibrinopeptides cleavage by thrombin. Data were analyzed with the Protein Solutions DynaPro Dynamics 6.2.04 software (Rheometric Scientific, Munchen, Germany), using the Cumulants method to fit the autocorrelation functions, where the uniformity of sizes is determined through a monomodal (single particle) curve fit analysis assuming a single particle size with a Gaussian distribution [14]. The program also reports the SOS (Sum Of Squares) difference between the measured and the Cumulants calculated intensity correlation curves, which is reported for each sample acquisition. Both the SLS and the DLS signals were processed, and, when applicable, averages over a series of measurements were made by weighting each data point by its $[1/(SOS)^2]$. The SLS data after averaging were also blank-subtracted and concentration-normalized, $[<I_{bs}>_w]_{wa,n} = [(<I_s>_w)_{wa} - (<I_b>_w)_a]/c$, where $(<I_s>_w)_{wa}$ is the SOS-weighted average intensity of the sample and $(<I_b>_w)_a$ is the arithmetic average intensity of a blank (TBS, for our

experiments 15,607 ± 271 counts/s; 24,558 ± 2508 counts/s with GPRP-NH_2/GHRP-NH_2 1.92 mM). The $[<I_{bs}>_w]_{wa,n}$ could then be converted into apparent weight-average molecular weights $[<M^*>_w]_{wa}$ (apparent because no angular- and concentration-dependence of the scattering signal are taken into account) using the Rayleigh-Gans-Debye [13] approximation:

$$\frac{1}{<M^*>_w} = K_v \frac{I_T\, n_T^2}{[<I_{bs}>_w]_{wa,n}\, R_{v,T}\, n_0^2 \sin(\vartheta)} \quad (1)$$

where K_v is the optical constant for vertically polarized light:

$$K_v = \frac{4\pi^2 n_0^2}{N_A \lambda^4}\left(\frac{dn}{dc}\right)^2 \quad (2)$$

with N_A Avogadro's number, λ the incident light wavelength *in vacuo* (824.8 nm), n_0 and dn/dc the TBS index of refraction and fibrinogen's differential refractive index increment at the operating temperature and λ, respectively, (1.3315 and 0.188 cm^3/mg, respectively, both extrapolated from data at lower wavelengths [5,15]). I_T is the measured intensity (131,040 ± 340 counts/s in our case) of neat toluene (Uvasol, Merck-Millipore) used as a reference scatterer (filtered with solvent resistant 0.2 μm pore-size filters; Gelman Acrodisc, Sigma-Aldrich), and n_T (1.4856) and $R_{v,T}$ (6.15×10^{-6}) are the refractive index and the Rayleigh ratio for vertically polarized light, respectively, of toluene, extrapolated at 20 °C and at λ = 824.8 nm from literature data [16,17], while θ is the scattering angle, here 90°. A calculated buffer (TBS) viscosity η = 1.0294 cP at 20 °C was used in the conversion from $<D_t>_z$ to $<R_s>_z$.

The measured $[<M^*>_w]_{wa}$ were then extrapolated to c = 0, $[<M^0>_w]_{wa}$, using the relation:

$$\frac{1}{<M^*>_w} = \frac{1}{<M>_w}\left(1 + 2BM_1 c\right) \quad (3)$$

where BM_1 = -84.1 g/mL is the second virial coefficient of HMW-FG determined by sedimentation velocity analytical ultracentrifugation by Raynal et al. [6].

Hypothetical αC-αC-mediated HMW-FG and DD-FG complexes and statistical evaluation against experimental SLS data.

Following thrombin removal of the fibrinopeptides and in absence of knob-hole interactions (defective knobs in DD-FG, holes blocked by synthetic knobs mimics in HMW-FG), the amount of hypothetical αC-αC-mediated complexes was evaluated as a function of FG species concentration as described in [18], using the experimental K_d for the recombinant human fibrinogen αC-region (α392-610), 12 μM [19].

We start with:

$$K_d = \frac{[\mathrm{M}]^2}{[\mathrm{D}]} \tag{4}$$

and

$$[\mathrm{FB}]_\mathrm{T} = [\mathrm{M}] + 2[\mathrm{D}] \tag{5}$$

where [M] and [D] are the molar concentrations of monomeric and dimeric fibrin species, respectively, and $[\mathrm{FB}]_\mathrm{T}$ is the total molar fibrin species concentration. Substituting and rearranging, we have:

$$K_d = \frac{2[\mathrm{M}]^2}{[\mathrm{FB}]_\mathrm{T} - [\mathrm{M}]} \tag{6}$$

from which [M] is determined as:

$$[\mathrm{M}] = \frac{-K_d + \sqrt{K_d^2 + 8\,[\mathrm{FB}]_\mathrm{T}\,K_d}}{4} \tag{7}$$

The SLS-determined $[<M^0>_w]_{wa}$ values, corrected for the small loss of mass following cleavage of the fibrinopeptides, $([<M^0>_w]_{wa})_{\text{-Fps}}$, were used to determine the $[\mathrm{FB}]_\mathrm{T}$ values from the effective concentration c_{eff} of the FB species bearing the potential αC-region interaction domain α1-503, corrected for the dilution upon adding thrombin:

$$c_{\mathrm{eff}} = c_{\mathrm{meas}} \times [\%(\alpha 1\text{-}503)/100] \times (V_\mathrm{i} \times c_{\mathrm{meas}} / V_\mathrm{f}) \tag{8}$$

$$[\mathrm{FB}]_\mathrm{T} = c_{\mathrm{eff}} / ([<M^0>_w]_{wa})_{\text{-Fps}} \tag{9}$$

where V_i and V_f are the sample volumes before and after thrombin addition, respectively.

We then computed what hypothetical $(<M>_w)_\mathrm{D}$ we would have determined by SLS for some discrete combinations of the weight fractions of monomers and dimers (w_M and w_D, with $w_\mathrm{M} + w_\mathrm{D} = 1$) in solution, including those predicted from the K_d calculations:

$$(<M>_w)_\mathrm{D} = \sum w_i M_i = w_\mathrm{M}\left(\left[<M^0>_w\right]_{wa}\right)_{\text{-Fps}} + w_\mathrm{D}\left[2\left(\left[<M^0>_w\right]_{wa}\right)_{\text{-Fps}}\right] \tag{10}$$

propagating the experimental uncertainties associated with the measured $[<M^0>_w]_{wa}$.
We could then compare these calculated $(<M>_w)_\mathrm{D}$ values with the measured $[<M^*>_w]_{wa}$ values obtained from SLS after prolonged thrombin treatment (see Table 2). To asses the

statistical significance between what we measured and what we would have measured if a certain % of dimers had been formed, we used a one-tailed *t*-test after taking into account the correlation effects present in our SLS data series, using equations 10 and 12-14 of Zwiers and von Storch [20]. The correlation correction is necessary because our $[<M^*>_w]_{wa}$ values are weighted averages over many (>30) sequential SLS measurements (each lasting either 1, 10 or 20 s) taken on the same solution, and therefore the effective number of independent measurements is less than their total number, affecting the statistics. In short, for each associated series of measurements ($<I_s>_w$ SLS data before and after thrombin addition, hereafter indicated as the "*x*" and "*y*" sets) we began with an estimation of the lag-1 correlation coefficient r_1 as:

$$r_1 = \frac{\sum_{t=2}^{m}(x_t - \bar{x})(x_{t-1} - \bar{x}) + \sum_{t=2}^{n}(y_t - \bar{y})(y_{t-1} - \bar{y})}{\sum_{t=1}^{m}(x_t - \bar{x})^2 + \sum_{t=1}^{n}(y_t - \bar{y})^2} \qquad (11)$$

where *m* and *n* are the number of measurements for sets *x* and *y*, respectively, *t* is the nth measurement in a series, and $\bar{x}$ and $\bar{y}$ are the mean of the $(<I_S>_w)_t$ values of the two series. Next, the equivalent sample size $\hat{n}'_e$ (and correspondingly $\hat{m}'_e$) is estimated as:

$$\hat{n}'_e = \begin{cases} 2 & \text{if } \hat{n}_e \le 2 \\ \hat{n}_e & \text{if } 2 \le \hat{n}_e \le n \\ n & \text{otherwise} \end{cases} \qquad (12)$$

with

$$\hat{n}_e = n(1 - r_1)/(1 + r_1) \qquad (13)$$

The pooled sample variance s^2 is instead calculated from the already computed SDs of the $(<M>_w)_D$ (predicted $<M>_w$ as a function of % dimers) and $[<M^*>_w]_{wa}$ (measured $<M>_w$ at the end of thrombin incubation) values:

$$s^2 = \left[\left(\mathrm{SD}_{(<M>_w)_D}\right)^2(m-1) + \left(\mathrm{SD}_{[<M^*>_w]_{wa}}\right)^2(n-1)\right] / (m+n-2) \qquad (14)$$

Finally, the Student's *t* statistics were then computed as:

$$t = \frac{[<M^*>_w]_{wa} - (<M>_w)_D}{s\left[1/\sqrt{\hat{m}'_e} + 1/\sqrt{\hat{n}'_e}\right]} \qquad (15)$$

for the various computed $(<M>_w)_D$ values. The $p < 0.05$ values were computed online at Social Science Statistics (https://www.socscistatistics.com/pvalues/tdistribution.aspx); for the degrees of freedom (DF), we used DF = $(\hat{n}'_e + \hat{m}'_e - 2)$. Additionally, calculations with DF = 2 are also reported as a very conservative test.

Sample preparation for SLS/DLS

LS techniques are very sensitive to monitor the early polymers formation following FG activation. However, this sensitivity requires very clean solutions. Anything that has a size comparable to or larger than the target molecule will also scatter light and confound the results (*w*- and *z*-means weight more any larger solute components than *n*- [number] means). For aqueous solutions, this is mainly due to dust particles, so having an absolute clean scattering cell, and filtering all solutions through an at least 0.2 μm pore size filter is mandatory. For FG, the SE-HPLC step is also usually mandatory to remove oligomers and larger aggregates, hence the operations described in the main text.

Protocol:

1-Inside a clean-air hood with a height extension (Cleansphere CA100; Safetech Limited, apparently no longer existent) a vacuum-actuated single-cuvette washer (C1295; Sigma-Aldrich) is set-up on top of a 500 mL Erlenmeyer vacuum flask. The washer liquid-receiving cup is fitted with a holed rubber stopper through which a ~10 cm plastic tube is forced. The male end of a 3-way nylon valve is tightly fitted on the top of the plastic tube, with 28 mm diameter, 0.22 μm pore-size filters (Corning 431212; Sigma-Aldrich) mounted on the other two, female ports of the valve. The top filter is fitted with the barrel of a 20 mL polypropylene syringe, which can be filled with MQ water. A Protein Solutions DynaPro square quartz cuvette (10 × 10 mm external dimension, 3 mm path length, three optical windows of which one at 90°, 40 μL minimum sample volume) can be made dust-free by repeated washing with the filtered MQ water using in-house vacuum suction. The cuvette can then be dried by switching the 3-way valve to the horizontal port where the other filter lets in only clean air. The vacuum is then gently released, and the cell is then removed and quickly sealed with a piece of Parafilm, detached from its paper cover under the clean-air hood, using the protected side.

2-About 500 μL of TBS are spin-filtered (0.22 μm pore-size Spin-X; Sigma-Aldrich), and the filtrate is used to repeatedly clean, in the clean-air hood, a 100 μL Hamilton syringe, which is then kept there.

3-More TBS is spin-filtered inside the same spin filter system, which is then opened under the clean-air hood and ~80 μL loaded into the clean syringe.

4-50 μL of the TBS solution are injected through the Parafilm into the square cuvette. Another piece of Parafilm is placed above the first one to re-seal it.

5-The cuvette can then be moved into the cell holder of the DynaPro instrument to check for cleanliness (under our conditions, about 14,000 counts/s, no heavy spikes) and, if the

test is passed, to record some intensity/DLS blanks. Otherwise, the washing procedure is repeated.

6-If the cuvette passes the test, the blank absorbance spectrum (250-350 nm) is recorded in the DU-640 spectrophotometer described in the main text.

7-The cuvette is then re-transferred under the clean-air hood.

8-A required amount of the sample solution is loaded into the spin-filter system which was already used for the TBS solution, and whose receiving end, emptied under the clean-air hood, was then kept clean. After spinning, the Spin-X is transferred and opened inside the clean-air hood.

9-The DynaPro cuvette under the clean-air hood is emptied using the clean syringe, through the Parafilm seal, discarding the content.

10-The spin-filtered sample is then loaded into the clean syringe in the clean-air hood, and 50 μL are injected through the Parafilm seal in the cuvette. Another small piece of Parafilm is placed over.

11-The cuvette is re-transferred to the DynaPro to check again for cleanliness and signal strength. If all is good, a set of intensity/DLS data are collected, and then the cuvette is removed and the absorbance spectrum of the sample is measured.

12-For activation with thrombin experiments, the cuvette is re-transferred under the clean-air hood.

13-Two other Spin-X filters are loaded with TBS, spun and transferred under the clean-air hood. The receiving end of the first is emptied of the filtered TBS and the FG content of the DLS cuvette is transferred inside it using the clean syringe (assuming no volume losses, i.e. 50 μL of FG solution). The receiving end of the other is emptied as well, cleaning in the process the syringe of any FG remains, and then a thrombin solution with the appropriate NIHu/mL is filtered inside it under the clean-air hood using a 1 mL plastic syringe with a Millex GV PVDF 4 mm diameter, 0.22 μm pore-size low-protein binding filter (SLGV-R04-NL; Merck-Millipore). The first ~200-300 μL of the filtered thrombin solution are discarded. Since for the thrombin solution we could afford a much larger volume, we used this filtering system to avoid the possibility of thrombin being bound by the Spin-X filter.

14-About 20-30 μL of the syringe-filtered thrombin solution are loaded into the clean syringe, and then exactly 10 μL are left loaded into it.

15-The 10 μL of clean thrombin are rapidly injected into the clean Spin-X receiving tube containing the 50 μL of FG solution and very briefly stirred with the syringe needle (a timer is started at this moment). The 60 μL are reloaded into the clean syringe and they are

rapidly re-injected into the DynaPro cuvette through the Parafilm, followed by the usual re-sealing. In certain cases 20 μL of thrombin solution were used in this activation procedure.
16-The cuvette is repositioned inside the DynaPro cuvette holder, and the measurements are started marking the time since activation (usually within 1-2 min since mixing began). Since sometimes heavy spikes can appear during a time course, the data collection can be stopped and restarted, keeping track of the time elapsed. In this way, multiple datasets can be collated to produce a full time-course.

Supporting Information References

1 Duval C, Allan P, Connell SD, Ridger VC, Philippou H, Ariens RA. Roles of fibrin α- and γ-chain specific cross-linking by FXIIIa in fibrin structure and function. *Thromb Haemost*. 2014; **111**: 842-50.

2 Biggs R. *Human Blood Coagulation, Haemostasis and Thrombosis*. Blackwell Scientific Publications / Lippincott, 1976.

3 Molteni M, Magatti D, Cardinali B, Rocco M, Ferri F. Fast two-dimensional bubble analysis of biopolymer filamentous networks pore size from confocal microscopy thin data stacks. *Biophys J*. 2013; **104**: 1160-9.

4 Gaffney PJ, Edgell TA. The International and NIH units for thrombin - How do they compare. *Thromb Haemost*. 1995; **74**: 900-3.

5 Cardinali B, Profumo A, Aprile A, Byron O, Morris G, Harding SE, Stafford WF, Rocco M. Hydrodynamic and mass spectrometry analysis of nearly-intact human fibrinogen, chicken fibrinogen, and of a substantially monodisperse human fibrinogen fragment X. *Arch Biochem Biophys*. 2010; **493**: 157-68.

6 Raynal B, Cardinali B, Grimbergen J, Profumo A, Lord ST, England P, Rocco M. Hydrodynamic characterization of recombinant human fibrinogen species. *Thromb Res*. 2013; **132**: e48-E53.

7 Higgins DL, Shafer JA. Fibrinogen Petoskey, a dysfibrinogenemia characterized by replacement of Arg-Aα16 by a histidyl residue. Evidence for thrombin-catalyzed hydrolysis at a histidyl residue. *J Biol Chem*. 1981; **256**: 12013-7.

8 Goldfarb AR, Saidel LJ, Mosovich E. The ultraviolet absorption spectra of proteins. *J Biol Chem*. 1951; **193**: 397-404.

9 Profumo A, Turci M, Damonte G, Ferri F, Magatti D, Cardinali B, Cuniberti C, Rocco M. Kinetics of fibrinopeptide release by thrombin as a function of $CaCl_2$ concentration: Different susceptibility of FPA and FPB and evidence for a fibrinogen Isoform-specific effect at physiological Ca^{2+} concentration. *Biochemistry*. 2003; **42**: 12335-48.

10 Wellings DA, Atherton E. Standard Fmoc protocols. *Method Enzymol*. 1997; **289**: 44-67.

11 Cardinali B, Lunardi G, Millo E, Armirotti A, Damonte G, Profumo A, Gori S, Iacono G, Levaggi A, Del Mastro L. Trastuzumab quantification in serum: a new, rapid, robust ELISA assay based on a mimetic peptide that specifically recognizes trastuzumab. *Anal Bioanal Chem*. 2014; **406**: 4557-61.

12 Anthis NJ, Clore GM. Sequence-specific determination of protein and peptide concentrations by absorbance at 205 nm. *Protein Sci*. 2013; **22**: 851-8.

13 Harding SE, Sattelle DB, Bloomfield VA. *Laser Light Scattering in Biochemistry*. Cambridge (UK): Royal Society of Chemistry, 1992.

14 Koppel DE. Analysis of macromolecular polydispersity in intensity correlation spectroscopy - Method of Cumulants. *J Chem Phys*. 1972; **57**: 4814-20.

15 Schulz GV, Ende HA. Über einige thermodynamische Eigenschaften von Fibrinogenlösungen auf Grund der Lichtstreuungsmethode. *Z Phys Chem*. 1963; **36**: 82-96.

16 Kedenburg S, Vieweg M, Gissibl T, Giessen H. Linear refractive index and absorption measurements of nonlinear optical liquids in the visible and near-infrared spectral region. *Opt Mater Express*. 2012; **2**: 1588-611.

17 Wu SJ, Huang SR. Optimal warranty length for a Rayleigh distributed product with progressive censoring. *Ieee T Reliab*. 2010; **59**: 661-6.

18 Benfield CT, Mansur DS, McCoy LE, Ferguson BJ, Bahar MW, Oldring AP, Grimes JM, Stuart DI, Graham SC, Smith GL. Mapping the IκB kinase κ (IKKκ)-binding interface of the B14 protein, a vaccinia virus inhibitor of IKKβ-mediated activation of nuclear factor κB. *J Biol Chem*. 2011; **286**: 20727-35.

19 Tsurupa G, Hantgan RR, Burton RA, Pechik I, Tjandra N, Medved L. Structure, stability, and interaction of the fibrin(ogen) αC-domains. *Biochemistry*. 2009; **48**: 12191-201.

20 Zwiers FW, Vonstorch H. Taking serial-correlation into account in tests of the mean. *J Climate*. 1995; **8**: 336-51.
